# Single-atom substitution redirects KatG reactivity from cofactor biogenesis to stereoselective sulfoxidation

**DOI:** 10.1038/s41467-026-73579-y

**Published:** 2026-06-13

**Authors:** Ran Duan, Jiasong Li, Wendell P. Griffith, Yang Xu, Nathan D. Burrows, Anthony P. Green, Aimin Liu

**Affiliations:** 1 Department of Chemistry, The University of Texas at San Antonio, San Antonio, TX 78249 USA; 2 Division of CryoEM and Bioimaging, Stanford Synchrotron Radiation Lightsource, SLAC National Accelerator Laboratory, Stanford University, Menlo Park, CA 94025 USA; 3 Manchester Institute of Biotechnology and Department of Chemistry, The University of Manchester, Manchester, M1 7DN UK; 4 Present Address: Key Laboratory of Agricultural Environmental Microbiology, Ministry of Agriculture, College of Life Sciences, Nanjing Agricultural University, Nanjing, 210095 P. R. China; 5 Present Address: Department of Biochemistry and Biophysics, and Department of Chemistry, University of Pennsylvania, Philadelphia, PA 19104 USA

**Keywords:** Oxidoreductases, Molecular conformation, Cryoelectron microscopy

## Abstract

Protein-derived cofactors rely on precisely positioned heteroatoms to direct redox chemistry, yet isolating their individual contributions remains challenging. The indole N–H of tryptophan plays a central yet elusive role in biogenesis and function of the Met–Tyr–Trp (MYW) cofactor in catalase-peroxidase (KatG). Here, we use genetic code expansion to replace cofactor-forming Trp105 with thiotryptophan (S-Trp), enabling a single-heteroatom (N → S) substitution. Instead of forming the MYW crosslink, KatG bearing S-Trp105 undergoes site-specific monooxygenation to yield a chiral sulfoxide. HPLC-MS, circular dichroism, and FT-IR spectroscopy identify selective oxygen insertion at the sulfur, establishing enantioselective formation of an (*S*)-configured sulfoxide. A 2.22 Å cryo-EM structure visualizes the oxidized S-Trp105, revealing the S = O moiety orienting toward the iron and confirming the absence of crosslinking. The S-atom oxygenation is heme-dependent and proceeds via a two-electron oxygen-atom transfer, contrasting with the radical-mediated one-electron chemistry of native tryptophan. This redirection suppresses catalase activity by perturbing cofactor formation. These results show that a single-atom substitution reroutes the distal heme site from radical crosslinking to stereoselective sulfoxidation, uncovering a monooxygenase-like capability within KatG. This work highlights using noncanonical amino acids to achieve atomic-level control over reaction pathways and to interrogate cofactor biogenesis with unprecedented precision.

## Abstract (web summary)

By swapping a single nitrogen atom for sulfur, researchers switched the enzyme KatG from its natural cofactor assembly to stereoselective sulfoxidation, revealing how atomic-level precision can reprogram complex enzymatic pathways.

## Introduction

Catalase-peroxidase (KatG) is a bifunctional enzyme broadly distributed across archaea, bacteria, and lower eukaryotes^1^. KatG combines a high-turnover catalase activity, catalyzing the disproportionation of hydrogen peroxide (H_2_O_2_) at rates of several thousand turnovers per second, with a slower peroxidase activity that oxidizes diverse substrates using H_2_O_2_ as the oxidant^2,3^. The catalase function critically depends on a protein-derived cofactor formed by a covalently crosslinked triad of methionine, tyrosine, and tryptophan side chains (MYW), whereas the peroxidase activity relies solely on the heme center^4^. In pathogenic organisms, the catalase activity of KatG is essential for survival within the host, where detoxification of H₂O₂ produced by the immune response is required for persistence and virulence^5,6^.

**Fig. 1:**
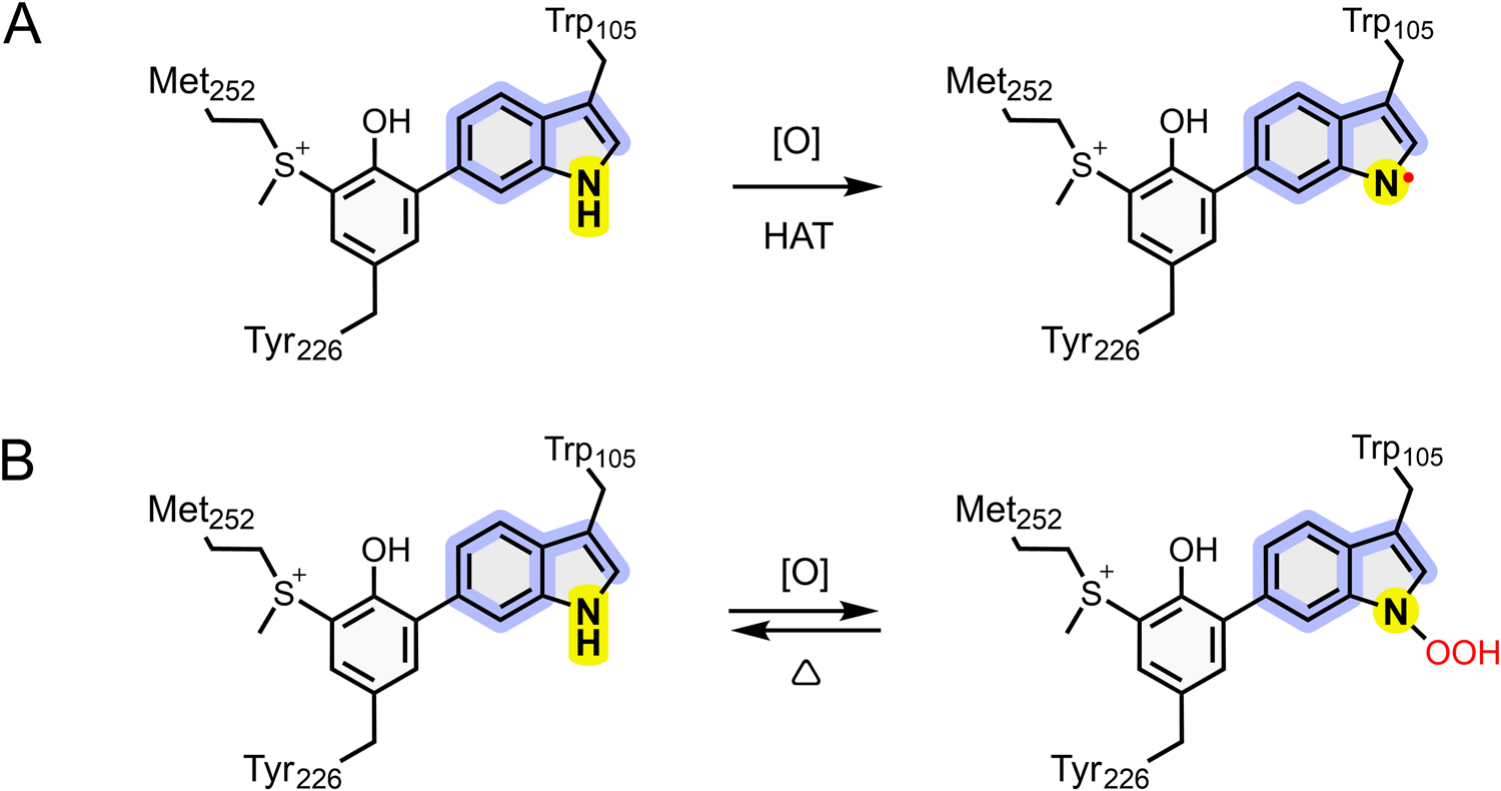
The indole N–H moiety of the protein-derived MYW cofactor in KatG is the critical oxidation hub for KatG reactivity. **A** The indole N-H moiety is proposed to be essential for hydrogen atom transfer (HAT) that elevates the MYW oxidation state and drives the catalase cycle. **B** The protein-derived cofactor in KatG is also naturally present in an oxygenated form with oxygen inserted into the indole N–H moiety, MYW-OOH, allowing the cofactor to enter a reversible, catalase-dormant yet primed state.

In contrast to monofunctional heme catalases, KatG employs the MYW cofactor to enable catalase activity through a protein-based radical mechanism (Fig. 1A). The MYW triad represents one of the most structurally and mechanistically intricate protein-derived cofactors known thus far, as its formation requires autocatalytic crosslinking of residues drawn from three distant regions of the polypeptide chain^7^. Elucidating the biosynthesis and function of MYW has proven challenging: cofactor formation occurs only once per polypeptide, generates weak and spectrally congested optical signatures, and is difficult to observe directly in intact protein^8^. Previous studies using heme-free KatG demonstrated that MYW formation can be initiated upon heme reconstitution followed by peroxide treatment, establishing that cofactor biogenesis is tightly coupled to heme-dependent oxidative chemistry^8,9^.

Structural and spectroscopic studies have revealed an alternative natural form of the triad, MYW–OOH, in which the indole nitrogen of tryptophan is peroxygenated (Fig. 1B). This species has been observed crystallographically in as-isolated KatGs from multiple organisms^6,10,11^ and, more recently, detected in solution as the predominant form of *Mycobacterium tuberculosis* (Mtb) KatG cultured under ambient conditions^11^. These observations highlight the functional importance of the indole N–H of tryptophan. Although the indole nitrogen is the closest heavy atom of the MYW cofactor to the heme iron, it is still more than 4 Å away. Its position and chemical properties therefore support a role in hydrogen atom transfer (HAT) steps^1,11–15^, which initiate the radical-mediated crosslink formation during MYW biogenesis (Supplementary Fig. 1)^8,9,16,17^.

During catalytic turnover promoted by KatG, the MYW catalytic triad is oxidized to the MYW^•^, via the indole nitrogen^5,6^, which is believed to be essential for catalase function, as it retains the oxidizing equivalent in the distal heme pocket, allowing it to oxidize the next H_2_O_2_ rather than oxidizing a peroxidatic donor via the radical propagation pathway^16^. Despite its centrality, direct experimental interrogation of the indole N–H group in these processes has remained elusive.

Traditional site-directed mutagenesis has been the primary strategy for studying protein-derived cofactors. However, this approach is intrinsically limited for autocatalytically formed crosslinks such as MYW. Substitution of any cofactor-forming residue typically abolishes cofactor formation entirely, yielding variants that report only on loss of function rather than on mechanistic detail^7^. As a result, protein-derived radical cofactors have been largely inaccessible to systematic structure-function interrogation at the atomic level. To overcome these limitations, genetic code expansion has emerged as a powerful strategy to introduce noncanonical amino acids (ncAAs) site-specifically into proteins^18^. We and others have shown that this approach enables controlled perturbation of autocatalytically formed cofactors, including Cys–Tyr dyads in iron- and copper-containing enzymes, providing mechanistic insights unattainable by canonical mutagenesis^19–22^.

Here, we apply genetic code expansion to KatG by replacing the MYW-forming tryptophan with the sulfur-containing analog thiotryptophan (3-[3-benzothienyl]-L-alanine, S-Trp)^23,24^. This single-atom NH → S substitution preserves aromaticity while altering hydrogen-bonding capacity and redox chemistry, offering a precise means to redirect oxidative reactivity at a critical catalytic position. Rather than yielding a simple loss-of-function variant, S-Trp substitution reveals an unexpected alternative reaction pathway that illuminates how KatG controls radical versus two-electron oxidation chemistry at the protein–heme interface.

## Results

### Single-atom substitution at Trp105 redirects KatG catalytic function

To interrogate how the cofactor-forming tryptophan contributes to KatG reactivity, we replaced Trp105 in *Escherichia coli* KatG (EcKatG) with thiotryptophan (S-Trp) using genetic code expansion. EcKatG was selected for its robust heterologous expression and well-characterized catalytic properties^25^. S-Trp closely mimics the steric and aromatic features of tryptophan, with a benzothiophene ring system in which sulfur replaces the indole nitrogen. Critically, this NH → S substitution preserves aromaticity while eliminating the indole N–H proton, thereby precluding hydrogen atom transfer from this position. This single-atom modification provides a precise means to redirect redox chemistry at Trp105 without grossly perturbing protein structure. Using a high-fidelity engineered pyrrolysyl-tRNA synthetase (PylRS)/tRNA pair specific for S-Trp^24^, we achieved efficient and site-specific incorporation of S-Trp at position 105 (Supplementary Fig. 2).

**Fig. 2:**
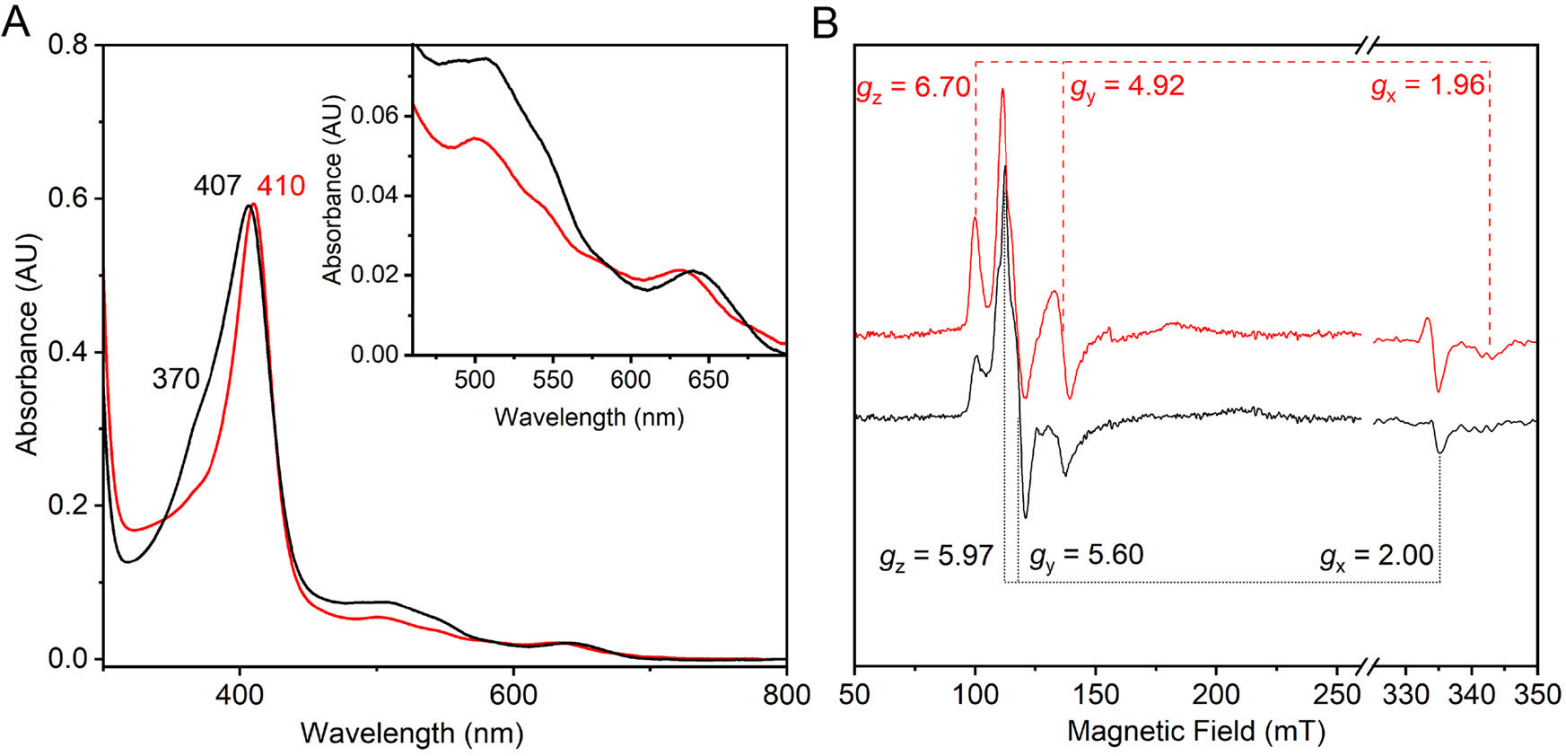
Substitution of the indole nitrogen with sulfur results in changes to the heme electronic environment. **A** UV-vis spectra of WT KatG (black) and KatG S-Trp105 (red) showing a red shift of the Soret band from 407 to 410 nm and attenuation of the visible-region absorbance feature. The insignificant differences of the α/β (Q-) bands are shown in an inset. **B** Comparison of the 10 K EPR spectra of WT KatG (black) and KatG S-Trp105 (red). Both spectra show a mixture of two high-spin ferric heme signals.

Although Trp105 is located several angstroms from the heme iron, substitution with S-Trp produces measurable changes in the optical properties of KatG. The S-Trp105 variant exhibits a red-shifted Soret maximum, 410 versus 407 nm in wild-type (WT) (Fig. 2A), and lacks the ∼370 nm shoulder observed in some KatGs and associated with cofactor-related electronic transitions^11^. In contrast, continuous-wave X-band EPR spectra of the S-Trp105 variant are essentially indistinguishable from those of the WT enzyme (Fig. 2B), indicating that the heme iron spin state and ligand field symmetry remain intact. Together, these data indicate that the NH → S substitution at Trp105 induces subtle but detectable perturbations of the heme electronic environment, detectable by UV-vis spectroscopy yet insufficient to alter the paramagnetic properties probed by EPR spectroscopy.

### Functional assays reveal that site-specific substitution of Trp with S-Trp profoundly alters KatG catalytic behavior

The S-Trp105 variant displays an approximately 10^3^-fold decrease in catalase activity relative to WT KatG (Table 1). It also exhibits lower activity than the Y226F variant, which is incapable of forming the MYW cofactor (Supplementary Fig. 3). Together, these observations indicate effective suppression of MYW‑dependent catalysis. Peroxidase activity is also reduced, with the S-Trp105 variant retaining approximately 6.3% of WT activity (Table 1). The magnitude and pattern of these changes are consistent with the effective suppression of MYW-dependent catalysis, while the specific retention of residual peroxidase activity prompted our investigation into the alternative reaction pathway characterized in the following section.

**Table 1:** Catalase and peroxidase kinetic parameters

Catalase activity	*k*_cat_ (s^−1^)	*K*_M_ (mM)	*k*_cat_/*K*_M_ (s^−1^M^−1^)
WT KatG	3900 ± 10	2.1 ± 0.2	(19 ± 2) x 10^5^
KatG S-Trp105	7.3 ± 0.2	8.3 ± 0.4	900 ± 50
KatG Y226F	13 ± 0.2	3.5 ± 0.2	3700 ± 400
**Peroxidase activity**	***k***_**cat**_ **(s**^**−1**^**)**	***K***_**M**_ **(mM)**	***k***_**cat**_**/*K***_**M**_ **(s**^**−1**^**M**^**−1**^**)**
WT KatG	0.63 ± 0.02	13 ± 1	48 ± 4
KatG S-Trp105	0.16 ± 0.01	51 ± 5	3 ± 0.3
KatG Y226F	4.20 ± 0.20	6.8 ± 1.1	620 ± 100

### Spectroscopic and mass spectrometric evidence for site-specific sulfur oxygenation

**Fig. 3:**
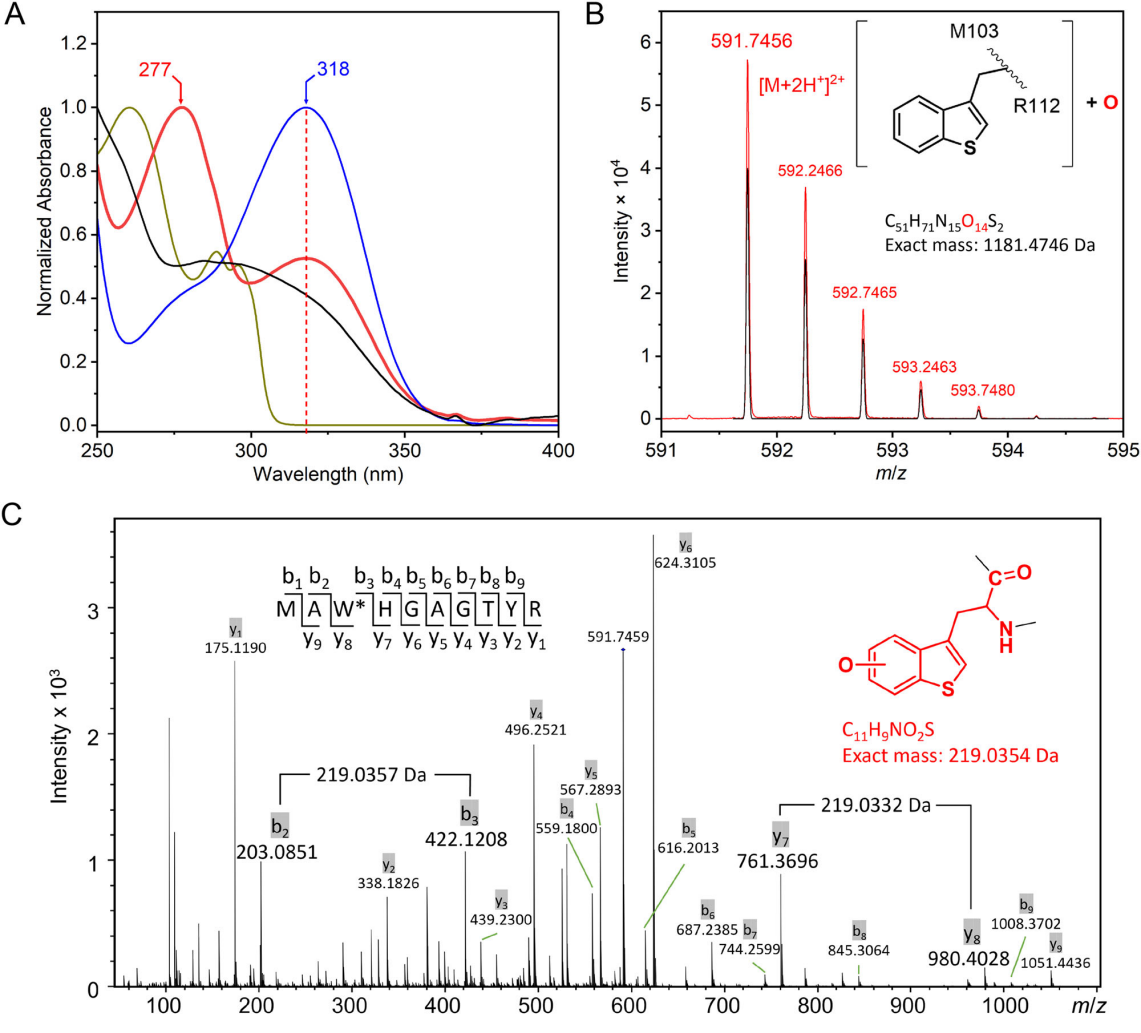
LC-MS analysis reveals autocatalytic oxidation of S-Trp in KatG. **A** UV-vis absorbance spectra of the peptide containing S-Trp105 (red), MYW-containing peptide from WT KatG (black), free S-Trp (dark yellow), and monooxygenated S-Trp standard (blue); all spectra are normalized to facilitate comparison. **B** High-resolution mass spectrum of the peptide bearing S-Trp105 (red), compared to the predicted isotopic distribution (black) **(C)** CID fragmentation spectrum of the S-Trp105 KatG-containing peptide; corresponding fragment ion assignments are provided in Supplementary Table 1.

Tryptic digests of S-Trp–incorporated KatG and the WT enzyme were analyzed by HPLC coupled with optical detection. As expected, WT KatG produced the characteristic Met–Tyr–Trp (MYW) crosslinked peptide fragment (CLPF), which exhibits a diagnostic absorption maximum at 330 nm and the predicted *m*/*z* value, eluting at a retention time consistent with previous reports for KatG cofactors (Supplementary Fig. 4)^9^. In contrast, no peptide corresponding to the MYW-containing CLPF was detected at this retention time in the S-Trp105 KatG digest (Supplementary Fig. 5). Instead, a distinct peptide eluted at 40.77 min and displayed absorption maxima at 277 and 318 nm (Fig. 3A), indicative of a chemically modified aromatic residue within the same sequence context.

The appearance of this peptide, together with the absence of the canonical MYW CLPF, indicates that substitution of Trp105 with S-Trp redirects cofactor biogenesis away from crosslink formation toward an alternative, site-specific chemical modification. These HPLC and optical spectral signatures are consistent with modification at position 105—now bearing a S-Trp residue or an oxidized derivative—rather than nonspecific degradation or loss of the cofactor-forming region, setting the stage for molecular identification of the oxidative product within S-Trp105 KatG.

High-resolution mass spectrometry (HRMS) was used to further characterize this peptide. The calculated monoisotopic mass of 1165.48 Da corresponds to residues 103 − 112: ^103^MAW*HGAGTYR^112^. The observed mass of 1181.47 Da (Fig. 3B) represents a + 15.99 Da shift, consistent with monooxygenation. Collision-induced dissociation (CID) fragmentation of the doubly charged ion (*m*/*z* 591.75) localized the oxidation to the S-Trp105 residue. The 219.0332 Da mass difference between b2/b3 and y7/y8 fragment ions matched the calculated mass of [S-Trp]-O (219.0354 Da) (Fig. 3B, Supplementary table 1). These findings demonstrate that the genetically encoded S-Trp105 undergoes monooxygenation during expression.

**Fig. 4:**
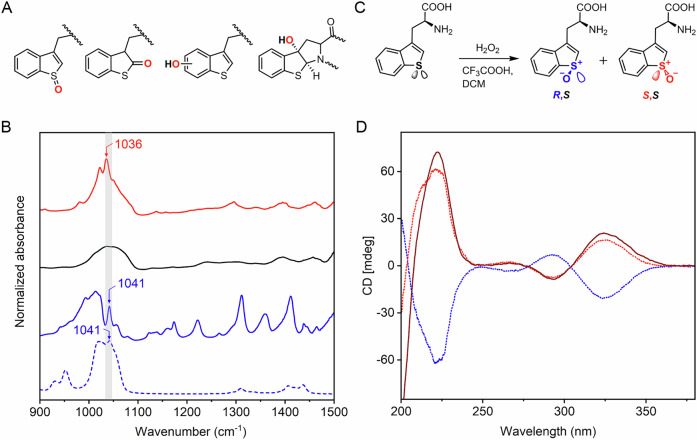
FT-IR and CD spectroscopies reveal regioselective sulfoxide formation and suggest a stereoselective S-oxygenation in KatG S-Trp105. **A** Chemical structures of potential monooxygenated and hydroxylated S-Trp species. **B** FT-IR spectra of lyophilized samples: WT KatG (black), KatG S-Trp105 (red), synthetic free O = S-Trp amino acid standard (solid blue), and DMSO (dashed blue). **C** Structures of two diastereomeric sulfoxides generated by non-enzymatic oxidation of S-Trp: (*R*,*S*) O = S-Trp and (*S*,*S*) O = S-Trp. **D** Circular dichroism spectra of free (*R*,*S*) (blue dots) and (*S*,*S*) (red dots) O = S-Trp amino acid standards, compared to the KatG peptide fragment that contains O = S-Trp105 (solid red trace). The peptide spectrum was smoothed using a Savitzky-Golay filter and normalized. Raw data are provided in Supplementary Fig. 11.

Given this modification, we investigated the site of oxygen incorporation. While sulfoxide formation is a common outcome of oxidative products of heme or non-heme iron enzymes, including hydroxytryptophan^26–28^, a benzothienopyrrole analogous to a pyrroloindole product^29^, or 2-oxindole derivatives^30,31^, were also considered (Fig. 4A). To determine whether oxygenation occurred at the sulfur atom of S-Trp105, FT-IR spectroscopy was used to probe for the diagnostic S = O stretching vibration in the 1000 − 1100 cm^−1^ region^32,33^. Control samples included WT KatG, sulfoxide-modified S-Trp (O = S-Trp) free amino acids in (*R*)- and (*S*)-configurations, and dimethyl sulfoxide (DMSO). The S-Trp105 KatG variant exhibits a pronounced absorption band at 1036 cm^−1^ that is absent in WT KatG (Fig. 4B). This feature closely matches the S = O stretching frequencies observed for free O = S-Trp and DMSO ( ~ 1041 cm^-1^), providing strong spectroscopic evidence for site-specific sulfoxide formation at Trp105.

### Chiroptical signatures reveal enantioselective formation of a chiral S-Trp sulfoxide

Having unambiguously established sulfur oxygenation at Trp105, we next asked whether this oxidation is stereoselective within the KatG active site. Although the benzothiophene ring of S-Trp is intrinsically planar, monooxygenation at the sulfur atom generates a stereogenic center, giving rise to two possible sulfoxide enantiomers. In a protein environment such as KatG, where the modified residue is conformationally constrained and oriented relative to the heme center, oxygen atom transfer is expected to occur preferentially from one face of the sulfur atom. Such spatial control provides a basis for enzymatic stereoselectivity, potentially yielding a dominant sulfoxide configuration rather than a racemic mixture.

To establish chiral reference standards, free O = S-Trp was synthesized following a published procedure^34^, yielding two separable diastereomers, designated P1 and P2. The two isomers exhibited identical UV-vis spectra (Supplementary Fig. 5) and indistinguishable mass spectra (Supplementary Figs. 6, 7, and 9), while showing subtle but reproducible differences in their NMR profiles (Supplementary Figs. 8 and 10). Circular dichroism (CD) spectroscopy revealed mirror-image spectra for P1 and P2 (Fig. 4C), confirming that the two species share the same configuration at the α-carbon but differ in stereochemistry at the newly formed sulfur center (Fig. 4D).

To determine the stereochemical outcome of S-Trp oxidation within KatG, we next recorded the CD spectrum of the isolated S-Trp–containing peptide. Strikingly, its CD profile closely matches that of synthetic diastereomer P2 (Fig. 4D; Supplementary Fig. 11), indicating that oxygenation occurs selectively at a specific face of the sulfur atom. This correspondence establishes that sulfoxidation in KatG is highly stereoselective rather than stochastic. Moreover, the observed CD signature closely resembles that reported for (1 *R*)−3-methylbenzo[b]thiophene 1-oxide in the 200–400 nm region^35^, supporting assignment of the oxidized residue in KatG as the (*S*)-configured sulfoxide, O = S-Trp105.

### Cryo-EM reveals atomic-level structural redirection to an (*S*)-configured S-Trp sulfoxide

**Fig. 5:**
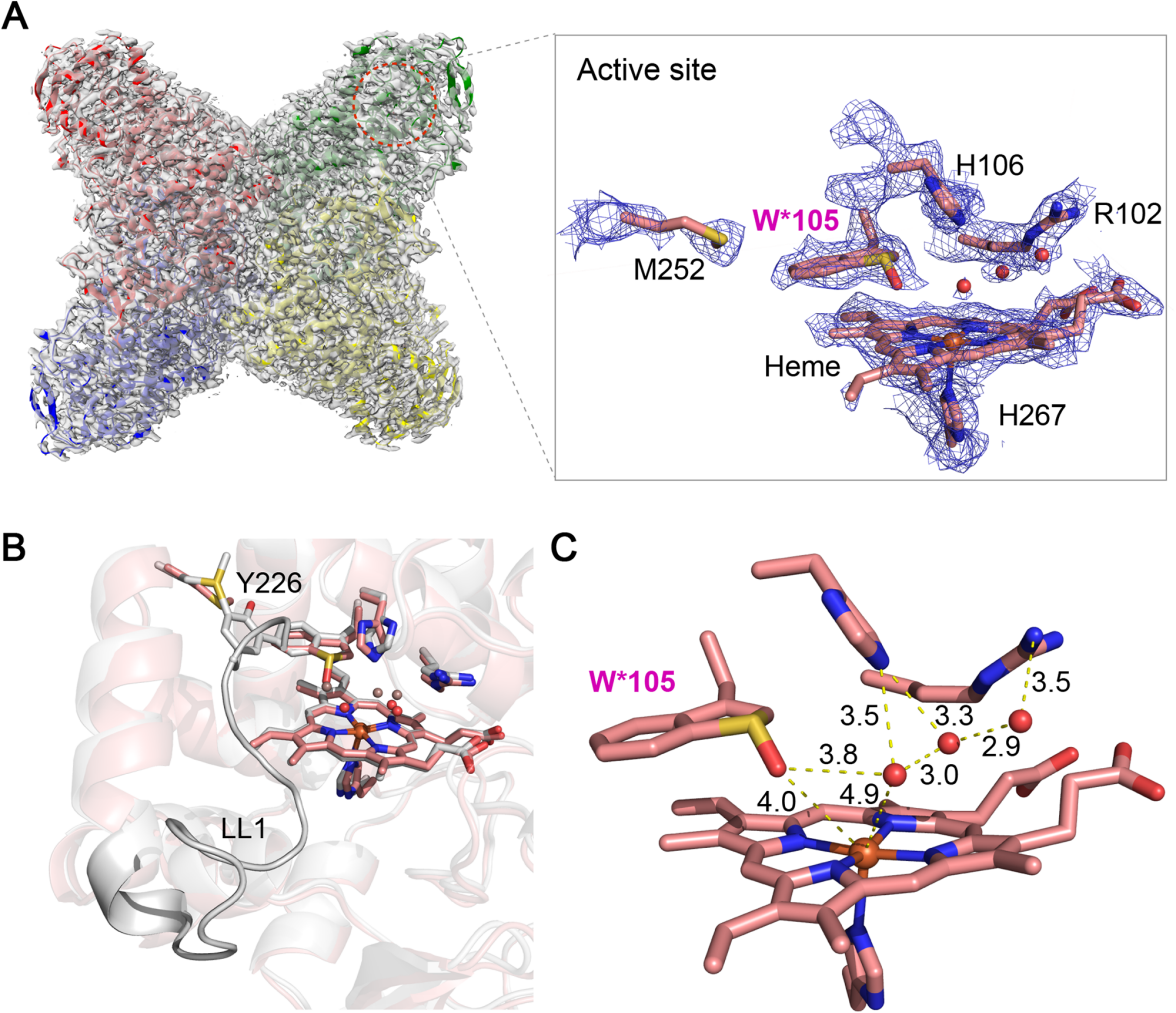
CryoEM structure of KatG S-Trp105 reveals asymmetric sulfoxide formation oriented toward the heme and absence of crosslinking. **A** Cryo-EM map and atomic model of tetrameric KatG S-Trp105 at 2.22 Å resolution (density modified; raw map resolution 2.29 Å); each protomer is shown in a distinct color (PDB entry: 9O6A)^58^. **A** close-up view of the active site shows the cryo-EM density and model fit. The sulfoxide adopts the (*S*) configuration, consistent with the observed asymmetric density at S-Trp105. **B** Structural overlay of KatG S-Trp105 (salmon, this study) and WT KatG (gray, PDB: 7JZ6), showing an RMSD of 0.440 Å across 611 aligned Cα atoms. The LL1 loop is partially ordered in the WT structure but unresolved in the S-Trp105 variant. **C** Zoomed-in view of the heme center and distal pocket, highlighting key residues and interatomic distances (Å).

Previously reported MYW triad lacking variants apparently have large structural differences from the MYW-bearing or MYW-OOH-containing structures, and until now, no crystal structures of the MYW cofactor-free or partially crosslinked KatG mutants are available. The advance of cryo-EM technology has enabled the determination of a tryptophan mutant structure of Mtb KatG at 3.3 Å resolution, with the loop containing the MYW crosslink triad disordered, except for the introduced arginine^36^. Inspired by this prior work and through extensive optimization, we determined a high-resolution cryo-EM structure of full-length KatG bearing S-Trp at position 105 (Fig. 5). The structure was refined to 2.22 Å resolution (Supplementary Table 2, and Supplementary Fig. 12 for the cryo-EM workflow). The enzyme assembles as a D₂-symmetric tetramer, consistent with the oligomeric organization observed for WT KatG (Fig. 5A). The resulting density map is of sufficient quality to allow unambiguous visualization of the heme cofactor, surrounding conserved residues, and the modified S-Trp105 side chain within the active site (Fig. 5A).

**Fig. 6:**
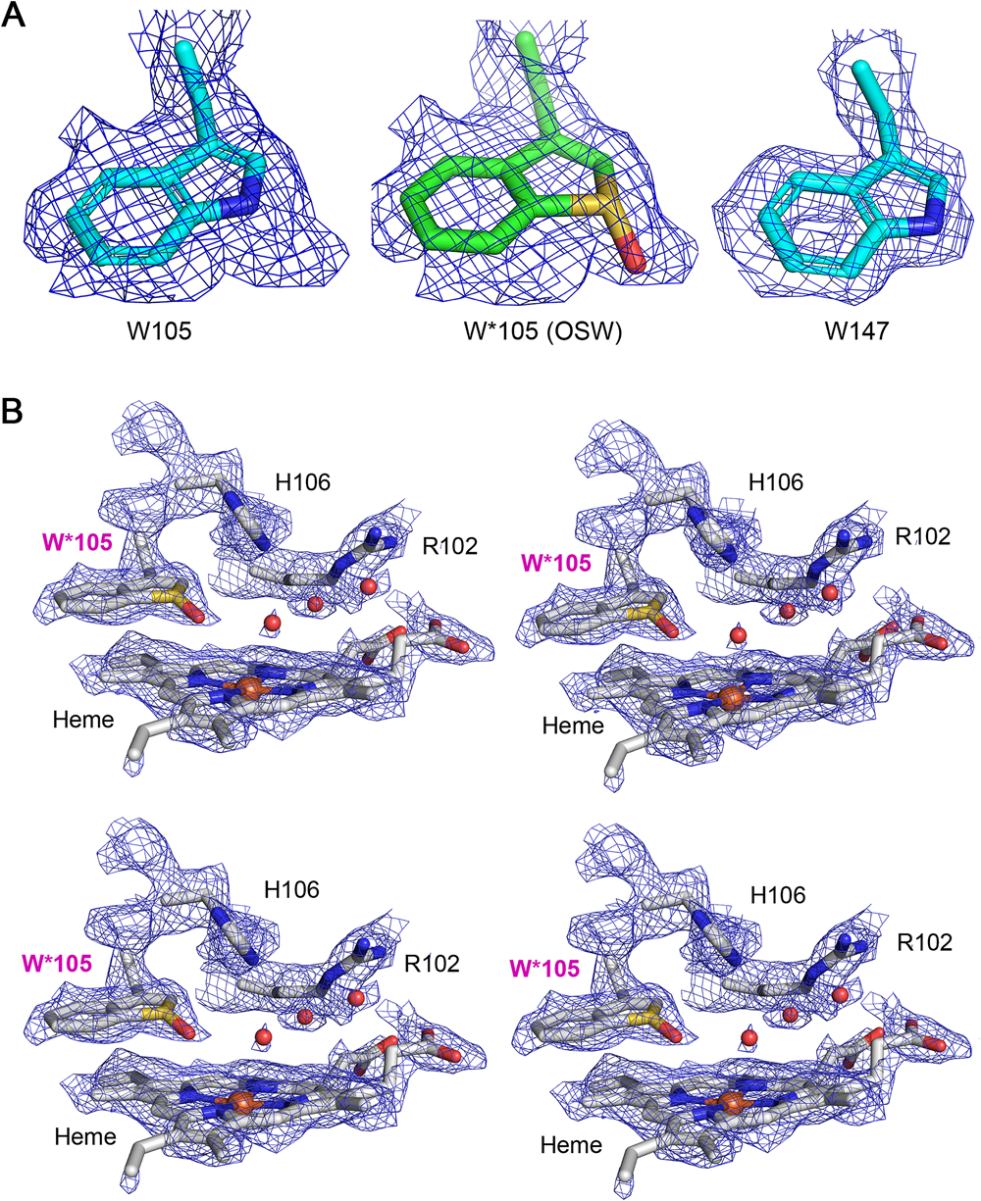
Stereogenic center of O = S-Trp105 in the cryo-EM structure of the engineered KatG variant. **A** Comparison of density map and model fitting on unmodified Trp, monooxygenated S-Trp (W*, molecular code in the 3D structure: OSW), and Trp147. Supplementary Fig. 14 shows the comparison of O = S-Trp electron density with all other Trp residues in the same KatG structure. **B** The (*S*)-configured aromatic sulfur atom-centered configuration is uniformly observed in all four chains of the asymmetric unit. The cryo-EM density map is contoured at 4.5 σ (ChimeraX).

The initial 2.29 Å raw map reconstruction exhibits a largely uniform local resolution distribution (Supplementary Fig. 13). Well-defined and continuous density is observed for the heme prosthetic group and key active-site residues, including Arg102, His106, Met252, and His267. The benzothiophene ring of S-Trp105 adopts an orientation closely matching that of Trp105 in WT KatG. Notably, an additional electron density, distinct from an unmodified Trp or S-Trp residue (Fig. 6A), is consistently observed at the sulfur atom of S-Trp105 in all four protomers (Fig. 6B), a feature absent at all other tryptophan positions (Supplementary Fig. 14). This density is fully consistent with the formation of a sulfoxide (S = O) moiety, corroborating the FT-IR and optical spectroscopic analyses. Modeling of O = S-Trp105 using geometric restraints appropriate for an *sp*^3^-hybridized sulfur atom (Supplementary Table 3) unambiguously favors the (*S*)-configuration at the sulfur center, with the sulfoxide oxygen oriented toward the heme iron. In contrast, an (*R*)-configured sulfoxide would direct the oxygen atom away from the heme and toward His106, a geometry incompatible with the observed density. This structural assignment independently confirms the stereochemical outcome inferred from CD spectroscopy.

Comparison with WT KatG structure reveals that substitution of Trp105 with S-Trp does not induce major rearrangements in the local active-site architecture. The side chain of O = S-Trp105 and neighboring residues (Arg102, His106, and Met252) remain closely aligned with their WT counterparts (Fig. 5A), indicating that the observed chemical redirection does not arise from global structural disruption. In contrast, pronounced differences are observed in the LL1 loop (Gly223–Ser240), which contains Tyr226, a constituent of the MYW cofactor.

In WT KatG, this loop is stabilized by covalent crosslinking and remains visible despite partial disorder^37^. In the S-Trp105 variant, however, a substantially expanded region of the LL1 loop (Trp197–Ser240) is unresolved in all four protomers (Fig. 5B), extending well beyond the disordered region observed in the WT enzyme. This loss of structural order is consistent with the absence of MYW crosslink formation and aligns with the spectroscopic, mass spectrometric, and functional data.

Taken together, the cryo-EM structure of KatG S-Trp105 provides direct, atomic-level evidence that a single-atom NH → S substitution redirects oxidative chemistry at the active site from radical-mediated crosslinking to stereoselective sulfoxidation (Fig. 6). The proximity of the sulfoxide oxygen to the heme iron (∼4.0 Å; Fig. 5C) offers a structural basis for the altered electronic coupling and the pronounced suppression of catalase and peroxidase activities observed for this variant.

### Two-electron oxygenation of S-Trp105 requires heme-dependent peroxide activation

To probe the mechanism underlying S-Trp105 oxidation, we generated a heme-deficient form of KatG S-Trp105 by culturing cells in iron-depleted medium. The resulting protein exhibited markedly reduced heme incorporation, as indicated by a low *R*_z_ value (0.080, compared to 0.361 for WT KatG), where the *R*_z_ value is defined as the ratio of the absorbance of the Soret band to the absorbance of the protein peak. Subsequent in vitro heme reconstitution yielded reconstituted EcKatG S-Trp105 (^R^EcKatG S-Trp105) with a substantially increased *R*_z_ value of 0.645 (Supplementary Fig. 15), confirming restoration of an intact heme center.

**Fig. 7:**
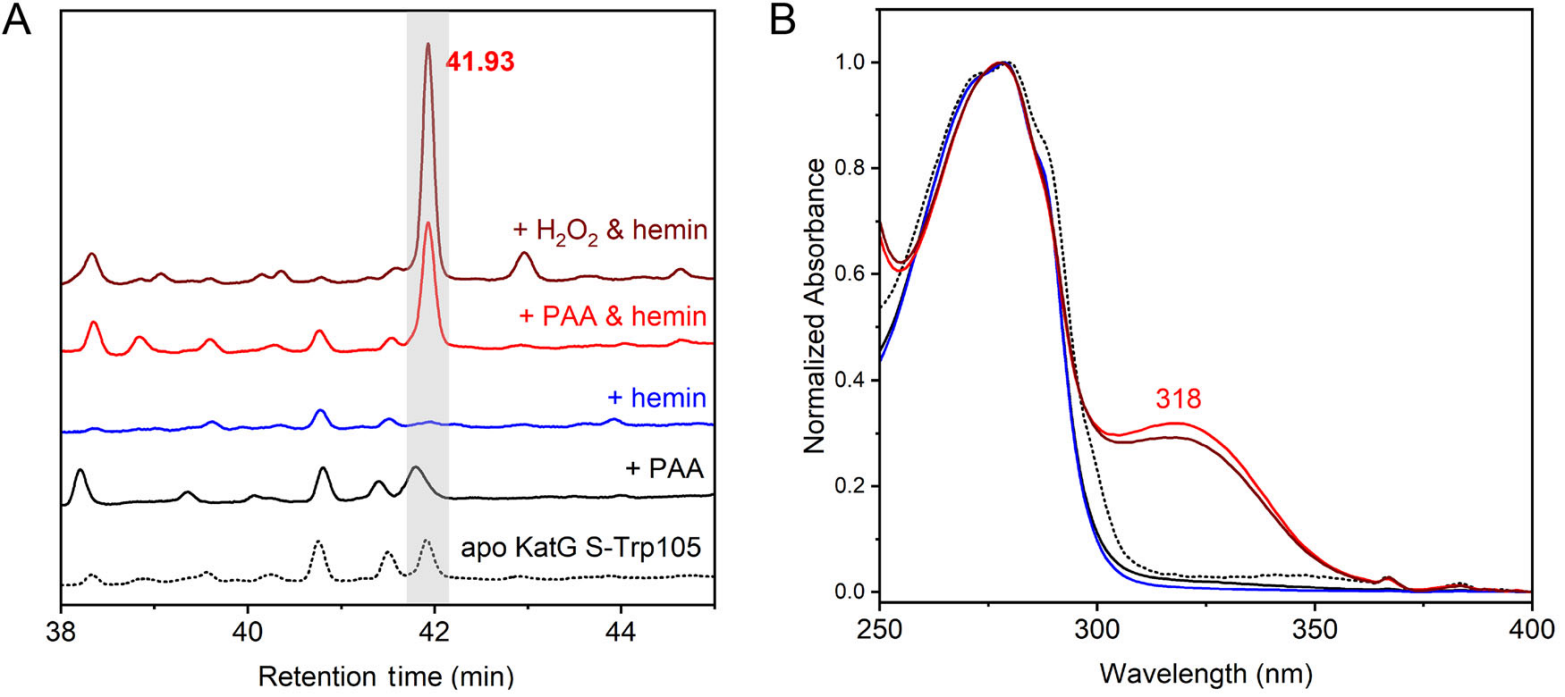
Heme and peroxide are required for S-Trp105 oxidation. **A** HPLC profiles of trypsin-digested samples: apo-KatG S-Trp105 treated with five equivalents of peracetic acid (black solid line), untreated apo-KatG S-Trp105 (black dashed), heme-reconstituted KatG S-Trp105 without oxidant (blue), and heme-reconstituted protein treated with peracetic acid (red) or H₂O₂ (magenta). **B** UV-vis spectra of HPLC fractions (retention time = 41.93 min). Only peptides from heme-reconstituted protein oxidized with peroxide exhibit the 318 nm absorbance peak characteristic of O = S-Trp.

To determine whether S-Trp105 oxygenation requires heme-mediated catalysis, both the heme-deficient and reconstituted S-Trp105 variants were treated with oxidants (5 equivalents of H_2_O_2_ or peracetic acid), followed by tryptic digestion and HPLC analysis. The formation of the oxidized S-Trp105 species, monitored by characteristic absorbance at 330 and 318 nm, was observed exclusively in the reconstituted enzyme (Fig. 7). No sulfoxide formation was detected in the heme-deficient protein under identical conditions. High-resolution mass spectrometry and CID analysis of the oxidized peptide (Supplementary Fig. 16, Supplementary Table 1) confirmed that it is identical to the O = S-Trp-containing peptide detected in the as-isolated S-Trp105 KatG (Fig. 3B).

These results demonstrate that S-Trp105 monooxygenation is strictly dependent on the presence of a functional heme center and an exogenous oxidant, establishing that sulfoxide formation is an active-site-mediated process rather than a nonspecific chemical modification. Together with the spectroscopic and structural data, these findings support a heme-dependent two-electron oxygen-atom transfer mechanism that autocatalytically converts the genetically incorporated S-Trp residue into a chiral sulfoxide in the mature enzyme. This pathway contrasts sharply with the radical-mediated chemistry of native Trp105 and underscores how a single-atom substitution redirects KatG reactivity.

## Discussion

Hydrogen atom abstraction (HAT) and transfer are fundamental to enzymatic redox catalysis, yet experimentally disentangling residue-specific contributions within complex active sites remains a longstanding challenge. In catalase–peroxidase (KatG), a heme-mediated HAT from the indole N–H of the cofactor-bearing Trp residue has long been proposed as the initiating step for both MYW cofactor biogenesis and catalase function^5,9,11,16,38–40^. However, direct experimental interrogation of the role of this specific proton has remained elusive. In this study, we show that substituting the indole N–H with sulfur at this single cofactor-forming position is sufficient to reroute the oxidative chemistry of KatG, demonstrating that the indole N–H is not merely permissive but obligate for enforcing the native radical pathway. By replacing Trp105 with thiotryptophan via genetic code expansion, we uncover an unexpected, site-specific, and stereoselective sulfoxidation catalyzed by the KatG heme center. This chemical redirection diverts oxidative flux away from the canonical one-electron, radical-mediated pathway responsible for MYW cofactor formation and instead reveals a two-electron oxygen-atom transfer process that is normally inaccessible in the native enzyme (Table 2).

**Table 2:** Single-atom substitution redirects catalytic pathways in KatG

Native tryptophan (N–H)	Thio-tryptophan (S)
Radical-initiated HAT pathway	HAT pathway inaccessible
Formation of Trp-centered radical (Trp-N^•^)	Two-electron aromatic S-oxygenation
MYW crosslink formation	Monooxygenase-like modification of an internal residue
Canonical catalase and peroxidase activities	Suppressed catalase activity and attenuated peroxidase activity

The engineered PylRS used in this study is specific in that it excludes the native Trp, indicating that it has achieved a high level of stereoelectronic complementarity for the sulfur atom. The PylRS active site is precisely tuned to the distinct bond lengths and polarizability of its cognate ncAA (S-Trp), highlighting the single-atom precision of this approach. The observed sulfoxidation is remarkable in several respects. First, it occurs on an internally encoded residue rather than an external substrate, demonstrating that KatG can catalyze monooxygenase-like chemistry within its own active site. Second, the reaction proceeds with regio- and stereo-selectivity, yielding the (*S*)-configured sulfoxide. Third, this chemistry is revealed not by altering the protein fold or active-site architecture, but by a minimal NH → S substitution that preserves aromaticity while fundamentally changing redox behavior. Together, these features indicate that KatG possesses an alternative chemical capability that is normally constrained by the chemical identity of Trp105.

**Fig. 8:**
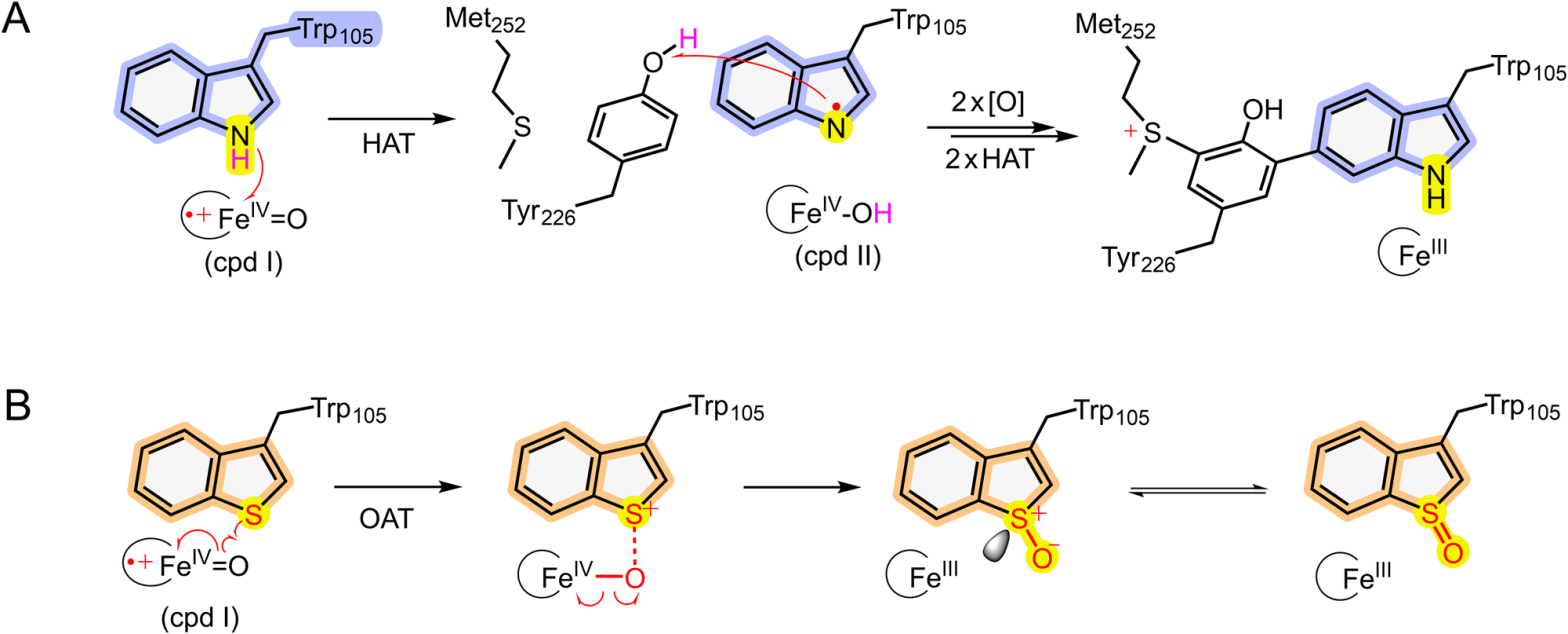
Single-atom substitution redirects KatG reactivity from one-electron, radical-mediated hydrogen atom transfer (HAT) chemistry for cofactor biogenesis to stereoselective sulfoxidation via a two-electron oxygen-atom transfer (OAT). Native pathway (Path **A**) proceeds via a radical-mediated mechanism initiated by an obligatory HAT from the indole N-H group to cpd I. This leads to the autocatalytic assembly of the Met–Tyr–Trp (MYW) crosslinked cofactor (for detailed arrow pushing of the biogenesis steps, see Supplementary Fig. 1). S-Trp variant (Path **B**) circumvents the radical path and instead facilitates a two-electron OAT. This direct oxidation, indicated by detailed arrow pushing, proceeds through a constrained ferryl (Fe^IV^) transition state. This interaction enhances facial selectivity, ultimately yielding the (*S*)-configured thiotryptophan sulfoxide while returning the heme to the ferric (Fe^III^) state.

The MYW crosslinking process has been hypothesized to begin with an HAT step on the indole N-H and three other HAT steps in two rounds of H_2_O_2_ oxidation (Fig. 8A, and for details see Supplementary Fig. 1)^8,9,16,17^. Experimental evidence for this biosynthetic sequence is difficult to obtain. Mechanistically, our data support a model in which removal of the indole N‑H group blocks the initial HAT for MYW biogenesis and thereby redirects oxidation to a two‑electron oxygen atom transfer (OAT) at the aromatic sulfur atom (Fig. 8B). The most plausible oxidant is Compound I (cpd I, i.e., ferryl and porphyrin π-cation radical). The strict dependence on an intact heme center and peroxide activation, together with the stereochemical outcome, argues against a radical-based mechanism and instead favors a constrained Fe–O···S transition state that enforces facial selectivity (Fig. 8B). Cryo‑EM observation of the sulfoxide oxygen oriented toward the heme iron provides a structural rationale for both the stereoselectivity and the pronounced perturbation of catalytic activity.

KatG’s ability to promote this transformation contrasts sharply with related heme peroxidases such as cytochrome *c* peroxidase (CcP) and ascorbate peroxidase (APX), where analogous active-site tryptophan residues are present and remain unmodified during catalysis^41,42^. This distinction likely reflects differences in oxidant reactivity and active-site dynamics: KatG favors highly reactive ferryl intermediates capable of driving oxygen-atom transfer, whereas CcP stabilizes a long-lived Compound ES (ferryl and an adjacent amino acid radical) state^43^. Consistent with this view, S-Trp substitution in CcP does not result in sulfoxidation despite the closer proximity of the sulfur atom to the heme center^24^. Thus, KatG’s distal pocket appears poised to enable controlled, enzyme-mediated aromatic sulfur oxidation.

An additional advance of this study is the use of high-resolution cryo-electron microscopy to visualize, at atomic detail, a site-specific post-translational modification of a genetically encoded noncanonical amino acid within an enzyme active site, even though this technology has previously been used to determine KatG structures^36,37^. The cryo-EM structure reported in this study reveals how local chemical modification propagates to disrupt MYW crosslink formation without inducing large-scale structural rearrangements. This represents a direct structural observation of enzyme-catalyzed sulfoxidation of an internally incorporated noncanonical residue.

More broadly, this work illustrates how ncAA substitution can be used not merely to test functional necessity but to actively redirect enzymatic reaction pathways and expose hidden catalytic capabilities. The distinct FT-IR and CD signatures of sulfoxide formation provide powerful spectroscopic handles for tracking regio- and stereochemical outcomes, suggesting that S-Trp may serve as a general probe for interrogating tryptophan-mediated redox chemistry. It is worth mentioning that genetic substitution of S-Trp into proteins is a recently developed technique, with prior efforts mainly aimed at probing the hydrogen-bonding roles of indole nitrogen in enzymes such as CcP, deubiquitinase, and thioredoxin^23,24,44^. Given the prevalence of indole and tryptophan in flavin- and metal–dependent oxidation systems, this strategy may find broad application in dissecting and reprogramming oxidative transformations across enzyme families.

Our results reveal the alternative chemical capability of the KatG active site and show how the chemical identity of the indole N–H proton channels reactivity into the native radical pathway. Active‑site tryptophan residues in heme peroxidases are intrinsically reactive: although residues such as Trp51 in CcP and the proximal Trp in APX often remain unmodified, perturbations in the distal pocket can promote one‑electron oxidation to give protein-based radicals or covalent heme-protein linkages. Examples, including the autocatalytic Trp51–Tyr52 crosslink in CcP mutants and the Tyr‑heme/Trp‑heme adducts observed in APX after peroxide treatment, illustrate that these residues are poised for oxidative transformation^45–47^. By contrast, the redirection of KatG chemistry to a site‑specific, two‑electron sulfoxidation of an internally encoded noncanonical residue represents a mechanistic departure from the one‑electron pathways described above (Fig. 8). Moreover, peroxidase-catalyzed enantioselective oxidations of free sulfides have been reported for various peroxidases, such as haloperoxidases, lignin peroxidase, and engineered dye-decolorizing peroxidases (DyPs)^48–54^, and human indoleamine 2,3‑dioxygenase (IDO) has been shown to perform monooxygenation on free S‑Trp^55^. However, the endogenous aromatic sulfide modification observed in S‑Trp KatG is distinct from these precedents. Rather than acting on an external substrate, the stereoselective sulfoxidation occurs within the KatG scaffold itself, illustrating how a single‑atom substitution can reprogram the enzyme’s internal maturation trajectory.

In summary, replacing a single heteroatom at Trp105 transforms KatG from a radical-based cofactor-forming enzyme into one that catalyzes stereoselective aromatic sulfoxidation. This atomic-level intervention reveals how enzyme function can be switched not by altering structure, but by redirecting chemistry—highlighting a powerful paradigm for probing and reengineering complex enzymatic redox processes.

## Methods

### Materials

The primers were purchased from Integrated DNA Technologies (IDT). All chemical reagents were purchased from Sigma-Aldrich or Thermo Fisher Scientific and were of reagent grade or higher and used as received. DNA manipulations in Escherichia coli (E. coli) were carried out according to standard procedures. Ampicillin (100 μg/mL) and chloramphenicol (30 μg/mL) were used as antibiotics to select recombinant strains.

### Generation of site-specific S-Trp-encoded KatG variant

EcKatG was chosen for this study due to its significantly higher protein expression efficiency compared to other available KatG expression systems. The katG gene was amplified from E. coli (K-12) genomic DNA and cloned into the pET-20b(+) vector containing a C-terminal six-histidine tag, which was developed in the Goodwin laboratory at Auburn University, Alabama, USA^25^. The resulting pET-20b(+)-EcKatG plasmid was used to transform an E. coli protein expression host (BL21 [DE3]), and transformants were selected based on their ampicillin resistance (100 μg/ml ampicillin). We generated W105TAG KatG mutant using the primers provided below:

5’- GGCCTAGCACGGCGCGGGGACTTACCGTTCAATCG −3’ and 5’-GCCGTGCTAGGCCATACGAATAAACAGACCGGCG-3’. Polymerase chain reaction (PCR) for generating the variants was performed using Phusion High-Fidelity polymerase with GC-buffer (Thermo Fisher Scientific). The PCR products were treated with DpnI to eliminate the template. Subsequently, E. coli DH10B was transformed with the DpnI-treated PCR products using a standard heat-shock procedure. Candidate plasmids were analyzed for complete sequence by Eurofins Genomics to verify the intended alterations. The pET-20b(+)-EcKatG_W105TAG plasmid was co-transformed into E. coli BL21(DE3) with the plasmid expressing a previously reported orthogonal tRNA/aaRS pair for S-Trp (pEVOL_PylRS_S-Trp plasmid)^24^, and the cells were plated onto an LB agar plate containing 100 μg/ml ampicillin and 30 μg/ml chloramphenicol.

### Protein expression and purification

The expression plasmid construct of EcKatG S-Trp105 was transformed into E. coli BL21(DE3), and transformants were selected based on resistance to chloramphenicol/ampicillin for EcKatG S-Trp105 expression. The bacteria were grown in an LB broth medium containing ampicillin (100 μg/ml) and chloramphenicol (30 μg/ml) in a baffled flask at 37 °C, 220 rpm. Arabinose, δ-aminolevulinic acid, and ferrous ammonium sulfate were added to a final concentration of 10 mM, 200 μM, and 25 μM, respectively. When the optical density at 600 nm (OD_600_) reached 0.4, isopropyl β-D-1-thiogalactopyranoside (IPTG) and S-Trp were added to a final concentration of 0.5 mM and 1 mM, respectively. After 12 − 14 h of incubation at 32 °C, 180 rpm, the cells were harvested and frozen at −80 °C for further use.

KatG S-Trp105 was purified by a fast protein liquid chromatography (FPLC) system. Cell pellets were resuspended in 100 mM sodium phosphate (NaPi) buffer (pH 7.0) with ~0.1 mM phenylmethylsulfonyl fluoride (PMSF) and then lysed by sonication. The supernatant was recovered upon centrifugation (34,000 × *g* for 40 min) at 4 °C and then applied to Ni-NTA agarose beads. After loading, the Ni-NTA agarose beads were washed with 2 column volumes of washing buffer (100 mM NaPi, 20 mM imidazole, pH 7.0). The His_6_-tagged protein was eluted with elution buffer (100 mM NaPi, 500 mM imidazole, pH 7.0). The eluted KatG S-Trp105 protein was further purified by a Superdex 200 (Cytiva) gel-filtration column (16/600) with 50 mM NaPi buffer (pH 7.0) as the mobile phase. The purified protein was either concentrated by ultrafiltration to the required concentration for subsequent experiments or stored in 50 mM NaPi, 5% glycerol buffer (pH 7.0) at −80 °C. The concentration of active heme protein was determined using the standard pyridine hemochromagen assay.

### Expression of apo-EcKatG S-Trp105 and heme-reconstitution

As previously described, the apo-EcKatG S-Trp (heme-free) was prepared by cell culture in iron-free medium^9^. All glassware was rinsed with 1 M HNO_3_ solution followed by deionized water (Millipore) before use. The iron-free medium was prepared by dissolving 10 g of casamino acids, 5 g of yeast extract, and 10 g of sodium chloride in 1 L of deionized water. Chelex 100 (30 g) resin was added to the medium and stirred for 2 h to remove trace metals. After filtration, the medium was transferred to a 2 L glass baffled flask and autoclaved. Supplemental ions and solutions, including 2 mM MgSO_4_, 0.1 mM CaCl_2_, vitamin mix (10 ml of a 100 mL stock: 10 mg each of riboflavin, niacinamide, pyridoxine monohydrate, and thiamine in 100 mL of H_2_O), and iron-free trace metals mix (10 mL of a 100 mL stock: 0.5 g of EDTA, 5 mg each of ZnCl_2_, CuCl_2_, CoCl_2_, and H_3_BO_3_, 160 mg of MnCl_2_, 50 mg of NiSO_4_, 100 ml of H_2_O), were added to the medium. The cell culture method was the same as the KatG S-Trp105, except that δ-aminolevulinic acid and ferrous ammonium sulfate were not added. The protein purification method was the same as for KatG S-Trp105.

Heme reconstitution for apo-EcKatG S-Trp105 was performed in 50 mM HEPES buffer (pH 8). A 5 mM stock solution of hemin was prepared by dissolving hemin into 50 mM NaOH solution. Hemin (1.5 molar equivalents relative to the protein) was added dropwise to the protein solution under stirring. The mixture was stirred at 4 °C for 2 h to allow complete hemin binding. The mixture was centrifuged at 20,000 × *g* for 5 min to remove any precipitated hemin and protein.

### Enzymatic activity and kinetics assay

Catalase activity was evaluated by measuring the O_2_ production rate using Oxygraph (Hansatech Pentney, Norfolk, England). The instrument was calibrated using sodium dithionite solution and air-saturated water. The reaction was initiated by adding 5 − 500 nM KatG enzyme to 0.25 − 25 mM H_2_O_2_ solution. All activity assays were carried out at 22 °C under 100 rpm stirring in 50 mM NaPi buffer (pH 7.0). The oxygen production speed was recorded and fitted with a hyperbolic curve using Origin software. Peroxidase activity was evaluated by monitoring the production rate of the 2,2’-azino-bis(3-ethylbenzothiazoline-6-sulfonic acid) (ABTS) radical (ε_417_ = 34.7 mM^−1^cm^−1^) using a Thermo Evolution Pro UV-vis spectrophotometer. The reaction was initiated by adding 5 to 500 nM KatG enzyme to 1 mM ABTS and 0.5 to 30 mM tert-butyl hydroperoxide (tBuOOH). All assays were carried out at 22 °C using 50 mM NaPi buffer (pH 7.0). Activity assays were performed in triplicate (three independent replicates). Data are presented as mean ± SD, and individual replicate values are shown as overlaid points on each plot.

### Trypsin digestion of protein and HPLC assay

Protein was diluted to a 2 mg/mL stock solution in 50 mM NH_4_HCO_3_ buffer (pH 8.5). MS grade trypsin protease (Fisher Scientific) was dissolved in the same NH_4_HCO_3_ buffer to a concentration of 1 mg/mL. A protein stock (100 μL) was mixed with 4 μL trypsin solution, leading to a 50:1 w/w ratio. The mixture was incubated at 37 °C for 48 h to ensure complete digestion. The digested protein samples were analyzed by SDS-PAGE to verify complete digestion. For the preparation and digestion of the ^R^EcKatG S-Trp105 sample, 50 mM HEPES buffer (pH 8.0) was used for digestion and heme reconstitution.

The digested protein was centrifuged at 20,000 × *g* for 5 min to remove any precipitate. A 100 μL sample was injected into an Inertsil ODS-3 HPLC column, 3 µm, 250 ×4.6 mm (GL Sciences) using an UltiMate 3000 HPLC (Thermo Scientific). The separation was performed using a linear gradient of buffer B (0.1% trifluoroacetic acid in acetonitrile) in buffer A (0.1% trifluoroacetic acid in water) from 0 − 60% over 2 h at a flow rate of 0.5 mL/min. Fractions with absorbance at 330 nm were collected and stored for MS analysis.

### Protein mass spectrometry

High-resolution electrospray ionization mass spectra (ESI-MS) were acquired using a maXis plus quadrupole-time-of-flight mass spectrometer (Bruker Daltonics) operated in positive-ion mode. Samples were directly infused from HPLC fractions into the ESI source using a syringe pump at a constant flow rate of 3 μL/min. Key source parameters were as follows: capillary voltage, 3500 V; nebulizer gas pressure, 0.4 bar; dry gas flow rate, 4.0 L/min; source temperature, 200 °C. CID was performed with an average collision energy of approximately 25 eV. Mass spectra were averages of 1-minute scans collected at a rate of 1 scan per second in the mass-to-charge ratio (m/z) range of 50 − 1500. Compass Data Analysis software version 4.3 (Bruker Daltonics) was used for all mass spectral data processing. Theoretical masses for b- and y-type fragment ions were calculated using the MS-Product utility of ProteinProspector (https://prospector.ucsf.edu/prospector/mshome.htm).

### Synthesis and separation of S-monooxygenated S-Trp (O = S-Trp)

Free O = S-Trp amino acid was synthesized following a modified published method^34^. Thiotryptophan (3-benzothienyl-L-alanine, or S-Trp) (221 mg, 1.0 equiv), 2 mL of dichloromethane, and 2 mL of trifluoroacetic acid were added to a round-bottom flask and cooled to 0 °C in an ice bath. An aqueous solution of H_2_O_2_ (0.88 mL, 30 wt%, 1.2 equiv) was added dropwise to the mixture. The flask was removed from the ice bath, and the mixture was stirred at room temperature for 1  h. The reaction was quenched with saturated NaHCO₃ solution and transferred to a separatory funnel. The organic layer was evaporated using a rotary evaporator, and the resulting solid was redissolved in water, combined with the aqueous layer, and lyophilized to yield an off-white powder. The solid powder was redissolved in 0.1% formic acid (FA) solution, filtered through a 0.22 μm filter, and separated by HPLC. For analytical HPLC, a 50 μL sample was injected onto an Inertsil ODS-3 HPLC column (3 µm, 100 × 4.6 mm, GL Sciences) using an UltiMate 3000 HPLC system (Thermo Scientific). Separation was achieved using an isocratic mobile phase of 30% acetonitrile, 0.1% FA, and 69.9% H₂O at a flow rate of 0.5 mL/min. For preparative HPLC separation, a 500 μL sample was injected onto a Hypersil PREP HS C18 column (5 µm, 250 × 10 mm, Thermo Scientific) using a preparative UltiMate 3000 HPLC system (Thermo Scientific). Fractions were separated using an isocratic mobile phase of 30% acetonitrile, 0.1% FA, and 69.9% H₂O at a flow rate of 3.5 mL/min. The collected fractions were lyophilized to a white powder and stored at −80 °C for future use.

### Optical and circular dichroism (CD) spectroscopies

UV-vis spectra were recorded at 22 °C in 50 mM NaPi buffer (pH 7.0), using a Thermo Evolution Pro UV-vis spectrophotometer. Fourier-transform infrared spectroscopy (FT-IR) spectra were collected using a Nicolet Nexus 470 FT-IR spectrometer with an ATR module. The spectra were acquired using lyophilized protein and commercially available chemicals. Circular dichroism (CD) spectra were recorded at 22 °C in deionized water using a Jasco J-1100 CD spectrometer under a continuous N_2_ purge with a cuvette path length of 1 mm.

### Electron paramagnetic resonance (EPR) spectroscopy

X-band continuous-wave EPR spectra were recorded on a Bruker E560 spectrometer equipped with a cryogen-free 4 K temperature system and an SHQE high-Q resonator, as described previously^56,57^. The measurements were performed at 9.37 GHz with 100 kHz modulation frequency, 1 mW microwave power, 0.6 mT modulation amplitude at 10 K, and an average of four scans for each spectrum. The EPR samples were 80 μM KatG protein (determined by heme concentration) in 50 mM NaPi buffer (pH 7.0) and were frozen in liquid nitrogen for storage.

### Cryo-EM sample preparation and data collection

Cryo-EM samples were prepared applying KatG S-Trp105 to grids at the Stanford-SLAC CryoEM Center (S^2^C^2^). KatG S-Trp105 protein samples were diluted to 1.5 μM in a 50 mM Tris-HCl and 50 mM NaCl buffer at pH 8.0. Grids were prepared using glow-discharged holey carbon grids (Quantifoil R2/1 Cu 200 mesh) and plunged into liquid ethane using a Thermo Fisher Scientific Vitrobot (Mk IV) cryo-plunger. Prior to sample application, grids were glow-discharged (PELCO easiGlow) at 15 mA for 30 s at 0.26 mbar residual atmosphere. A 3 µL aliquot of the sample was applied to each grid and blotted for 3 s under environmental chamber conditions of 100% humidity and 4 °C.

Cryo-EM data were collected at S^2^C^2^ using an Alpha Titan Krios G3i transmission electron microscope (TEM) (Thermo Fisher Scientific) operating at 300 keV, equipped with a Falcon 4i electron detector and a Selectris X energy filter (10 eV silt). Images were collected at a magnification of 130,000×, resulting in a pixel size of 0.954 Å per pixel, with an electron exposure of ~50 e^−^/Å^2^ at a dose rate of ~1.14 e^−^ per frame. The objective-lens defocus range was set between −1.0 and −2.0 μm.

### Cryo-EM data processing, Model building and refinement

Cryo-EM data processing was performed using cryoSPARC (Version 4.5.3). A total of 18,517 movies were imported. Following motion correction and contrast transfer function (CTF) estimation, 15,512 micrographs were retained for further analysis. Initial particle picking was performed using the blob picker with a particle diameter range of 100 − 220 Å. Particles were extracted from these micrographs with a box size of 240 pixels (downsized to 60 pixels by 4× binning), yielding 7,762,366 particles. These particles underwent three rounds of 2D classification, resulting in 2,821,979 particles. An ab initio reconstruction was then performed, sorting these particles into three volume classes.

To enrich rare-view particles, the particles from the best volume class were used as templates for template picking. This process generated 6,955,031 particles, which were then subjected to 2D classification, selecting 731,599 rare-view particles. These rare-view particles were combined with the previously retained particle set, resulting in a merged dataset of 3,060,258 particles. This combined set underwent heterogeneous refinement, and particle orientation was rebalanced, reducing the dataset to 884,126 particles from the best volume class. These particles were re-extracted with a box size of 240 pixels (unbinned) and further refined through sequential steps of homogeneous, non-uniform, and CTF refinement to produce the final reconstruction at 2.29 Å resolution.

The raw map from CryoSPARC was improved using the resolve_cryo_em job in Phenix (version 2.0rc1-5599), resulting in a density-modified map at 2.22 Å resolution. The initial template was from PDB 7JZ6 and docked into the map using phenix.dock_in_map. The model was refined using Coot (version 0.9.8.1) and phenix.real_space_refinement. The restraint of O = S-Trp was generated based on the geometry of *sp*^3^ hybridization and reference small-molecule structures (CCDC number 2236439). Statistics associated with data collection, 3D reconstruction, and model refinement are included in Supplementary Tables 2, and 3, and Supplementary Fig. 12.

### Reporting summary

Further information on research design is available in the Nature Portfolio Reporting Summary linked to this article.

## Supplementary information

Supplementary Information

Reporting Summary

Transparent Peer Review file

## Source data

Source File

## Data Availability

All data described in this study are included in the manuscript and the accompanying Supporting Information. The protein mass spectrometry datasets associated with Fig. 3 and Supplementary fig. 16 have been deposited in the ProteomeXchange Consortium via the PRIDE partner repository with the dataset identifiers PXD078006 for Fig. 2C and PXD078069 for Supplementary fig. 16, respectively. The cryo‑EM raw data generated in this study have been deposited in the Electron Microscopy Data Bank (EMDB) under accession number EMD‑70168 [https://www.ebi.ac.uk/emdb/EMD-70168], and the corresponding 3D coordinates have been deposited in the Protein Data Bank (PDB) under entry code 9O6A^58^. The PDB code of the previously published structure used in this study is 7JZ6. Source Data are provided as a Source Data file. Source data are provided with this paper.
