# Supplementary material for: Single-atom substitution redirects KatG reactivity from cofactor biogenesis to stereoselective sulfoxidation
Source: Nat Commun. 2026 Jun 13;17:5309. doi: 10.1038/s41467-026-73579-y (PMC13272816; doi:10.1038/s41467-026-73579-y)
Supplement: Supplementary file 1 — Supplementary Information [file 41467_2026_73579_MOESM1_ESM.pdf]

Supplementary Information

Single-atom substitution redirects KatG reactivity from cofactor biogenesis to stereoselective sulfoxidation

Ran Duan,[1] Jiasong Li,[1,4] Wendell P. Griffith,[1] Yang Xu,[2] Nathan D. Burrows,[2] Anthony P. Green,[3] and Aimin Liu[1,5,]✉

[1] Department of Chemistry, The University of Texas at San Antonio, San Antonio, TX 78249, USA

[2] Division of CryoEM and Bioimaging, Stanford Synchrotron Radiation Lightsource, SLAC National Accelerator Laboratory, Stanford University, Menlo Park, CA 94025, USA

[3] Manchester Institute of Biotechnology and Department of Chemistry, The University of Manchester, Manchester, M1 7DN, UK

[4] Current Address: Key Laboratory of Agricultural Environmental Microbiology, Ministry of Agriculture, College of Life Sciences, Nanjing Agricultural University, Nanjing, 210095, P. R. China

[5] Current Address: Department of Biochemistry and Biophysics, and Department of Chemistry, University of Pennsylvania, Philadelphia, PA 19104, USA

✉ Correspondence Author: Prof. Dr. Aimin Liu, E-mail: Feradical@utsa.edu

Table of Content

Supplementary figure 1. A proposed mechanism for MYW biogenesis involves four hydrogen atom transfer (HAT) steps on the indole N-H moiety in two rounds of heme-mediated H_2O_2 oxidation.

Supplementary figure 2. Purification of EcKatG S-Trp105

Supplementary figure 3. Catalase and peroxidase activities of WT, S-Trp105, and Y226F EcKatG proteins

Supplementary figure 4. HPLC and HRMS analysis of the MYW cofactor-bearing peptide in WT KatG

Supplementary figure 5. HPLC separation and UV-vis spectra of S-monooxygenated S-Trp diastereomers

Supplementary figure 6. High-resolution mass spectrum of fraction P3 in Supplementary figure 4 showing experimental and simulated (inset) isotope distributions for 2-amino-3-(1,1-dioxidobenzo[b]thiophen-3-yl)propanoic acid ($C_{11}H_{11}NO_4S$)

Supplementary figure 7. High-resolution mass spectrum of fraction P1 in Supplementary figure 4 showing experimental and simulated (inset) isotope distributions for 2-amino-3-(1-oxidobenzo[b]thiophen-3-yl)propanoic acid ($C_{11}H_{11}NO_3S$)

Supplementary figure 8. ^{1}H NMR (500 MHz, D_2O) and ^{13}C NMR (126 MHz, D_2O) spectra of fraction P1

Supplementary figure 9. High-resolution mass spectrum of fraction P2 in Supplementary figure 4 showing experimental and simulated (inset) isotope distributions for 2-amino-3-(1-oxidobenzo[b]thiophen-3-yl)propanoic acid ($C_{11}H_{11}NO_3S$)

Supplementary figure 10. ^{1}H NMR (500 MHz, D_2O) and ^{13}C NMR (126 MHz, D_2O) spectra of fraction P2

Supplementary figure 11. Original circular dichroism (CD) spectrum of the S-Trp105-bearing peptide isolated from digested KatG S-Trp105 via HPLC

Supplementary figure 12. Cryo-EM data processing workflow

Supplementary figure 13. Local resolution of the cryo-EM density map

Supplementary figure 14. Comparison of density map and model fitting on monooxygenated S-Trp (Molecular code in the structure: OSW) vs. unmodified Trp residues.

Supplementary figure 15. UV-vis and R_z value comparison of KatG S-Trp105, apo-KatG S-Trp105, and heme-reconstituted KatG S-Trp105

Supplementary figure 16. HRMS spectrum and CID spectrum for regenerated O=S-Trp bearing peptide

Supplementary table 1. Fragment assignment for the CID spectrum in Fig. 3C

Supplementary table 2. Cryo-EM data collection, processing, and refinement statistics

Supplementary table 3. The stereochemical restraints of O=S-Trp during cryo-EM data processing.

Supplementary table 4. Investigated potential heme-mediated autooxidation products in KatG S-Trp105

References Cited

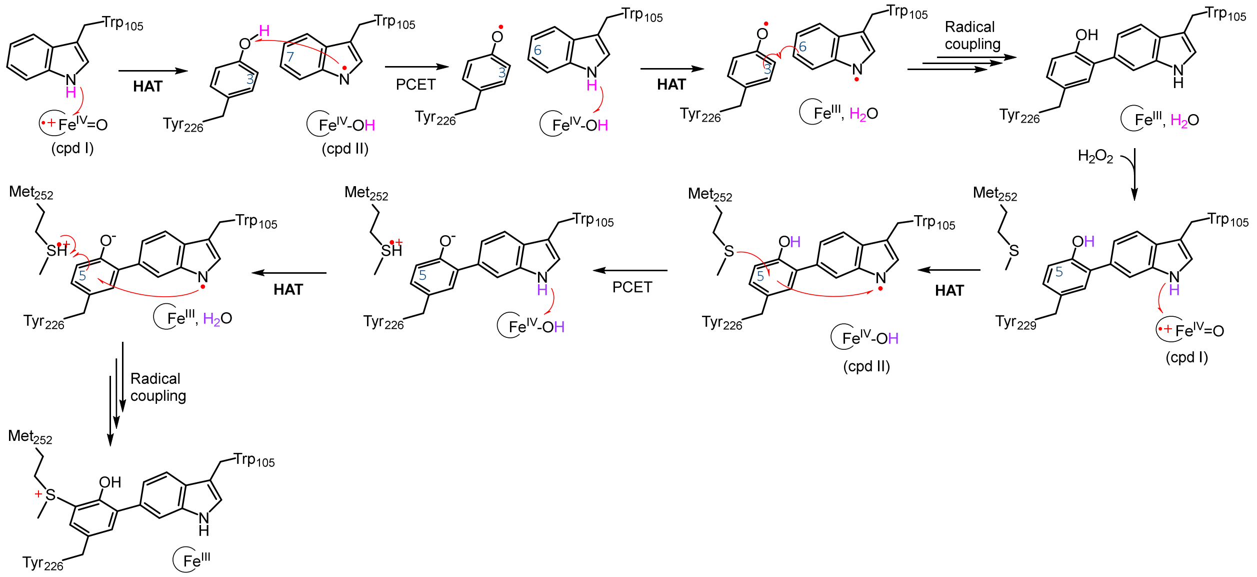

Supplementary figure 1. A proposed mechanism for MYW biogenesis involves four hydrogen atom transfer (HAT) steps on the indole N–H moiety in two rounds of heme-mediated H_2O_2 oxidation.

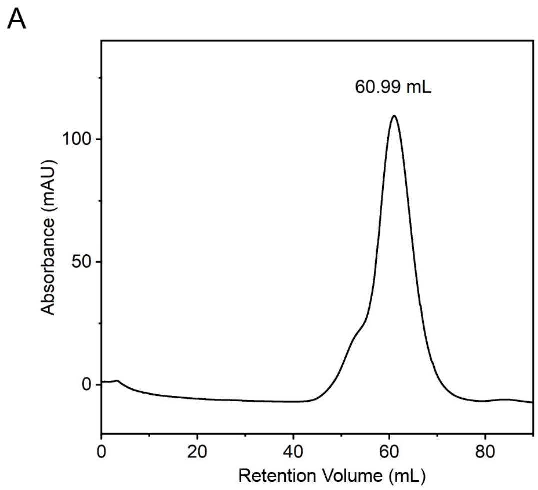

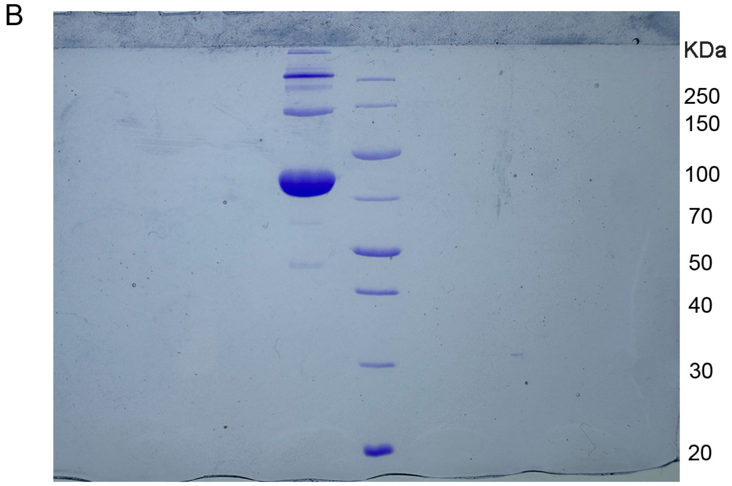

Supplementary figure 2. Purification of EcKatG S-Trp105. (A) Gel-filtration chromatography of as-isolated KatG S-Trp105 using a HiLoad 16/600 Superdex 200 column in 50 mM NaPi buffer (pH 7.0) at a 1.5 mL/min. (B) SDS-PAGE analysis of purified KatG S-Trp105 protein. The molecular weight markers are indicated on the right.

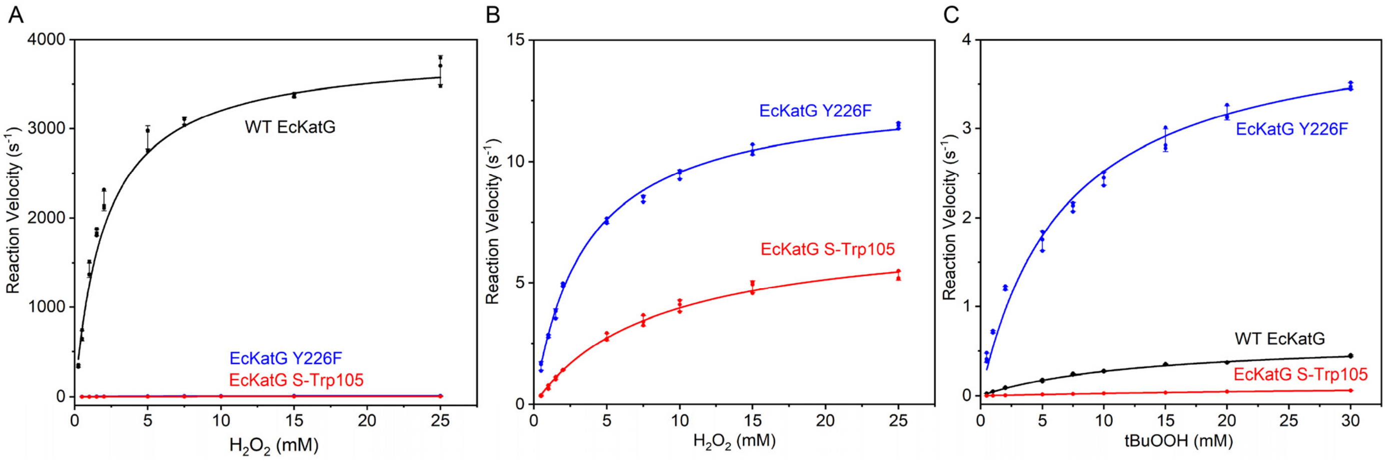

Supplementary figure 3. Catalase and peroxidase activities of WT, S-Trp105, and Y226F EcKatG proteins. (A) Catalase activity comparison. (B) Magnified view of catalase activities for KatG S-Trp105 and KatG Y226F. (C) Peroxidase activity comparison. Activities for WT (black), KatG S-Trp105 (red), and KatG Y226F (blue) are shown. Individual data points are overlaid on each bar. Error bars represent mean ± SD (n = 3 independent technical replicates).

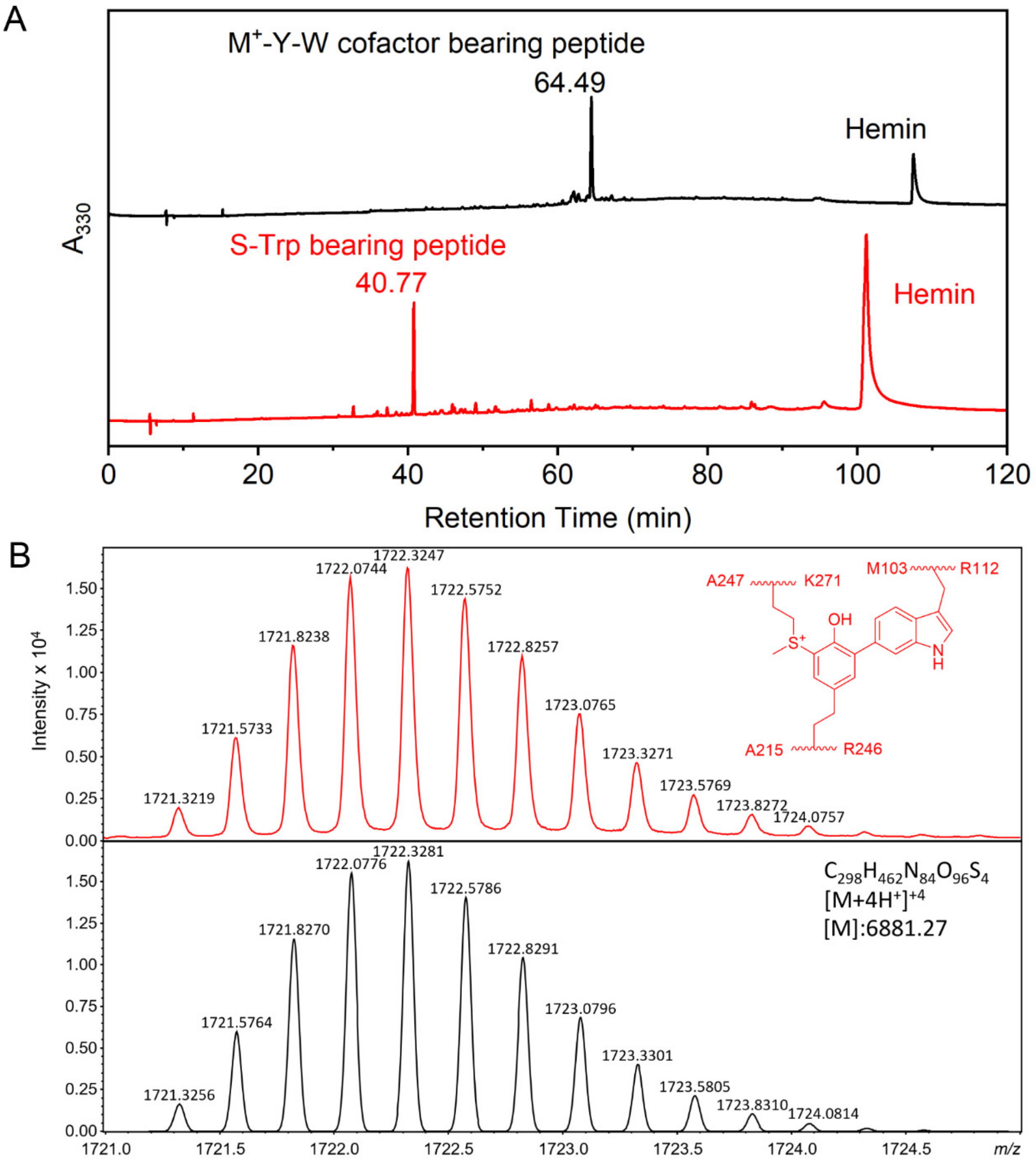

Supplementary figure 4. HPLC and HRMS analysis of the MYW cofactor-bearing peptide in WT KatG. (A) Comparison of HPLC profiles for the MYW cofactor peptide in WT KatG (black) and the corresponding S-Trp-bearing peptide in KatG S-Trp105 (red), highlighting their distinct retention times. (B) High-resolution mass spectrum (HRMS) of the MYW cofactor-bearing peptide from WT EcKatG (red) overlaid with the simulated spectrum (black).

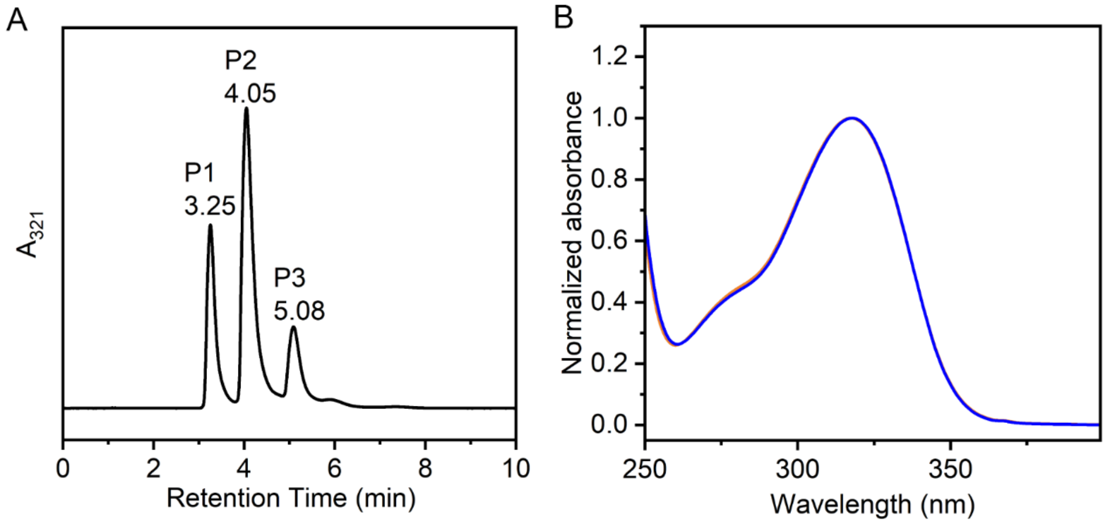

Supplementary figure 5. HPLC separation and UV-vis spectra of S-monooxygenated S-Trp diastereomers. (A) Preparative HPLC chromatogram showing the separation of S-monooxygenated S-Trp products (P1 and P2). (B) Comparison of UV-vis spectra for fractions P1 (orange) and P2 (blue).

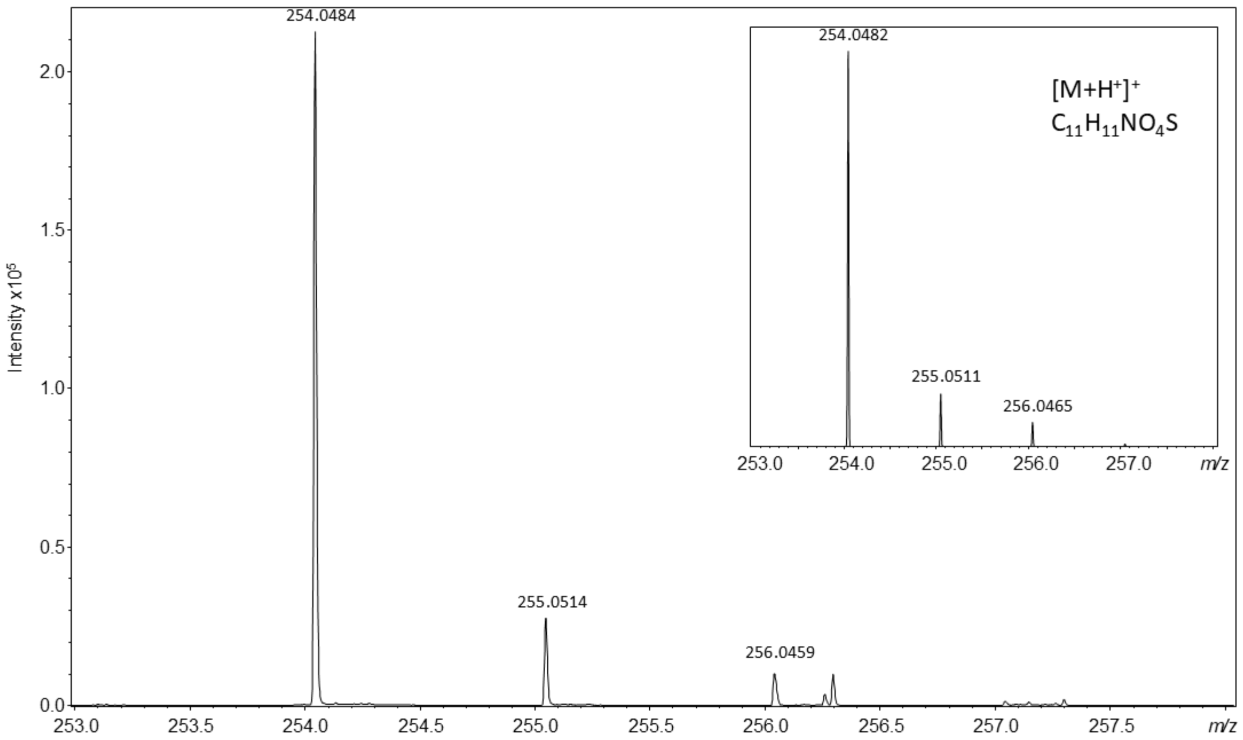

Supplementary figure 6. High-resolution mass spectrum of fraction P3 in Supplementary figure 5 showing experimental and simulated (inset) isotope distributions for 2-amino-3-(1,1-dioxidobenzo[b]thiophen-3-yl)propanoic acid ($C_{11}H_{11}NO_4S$).

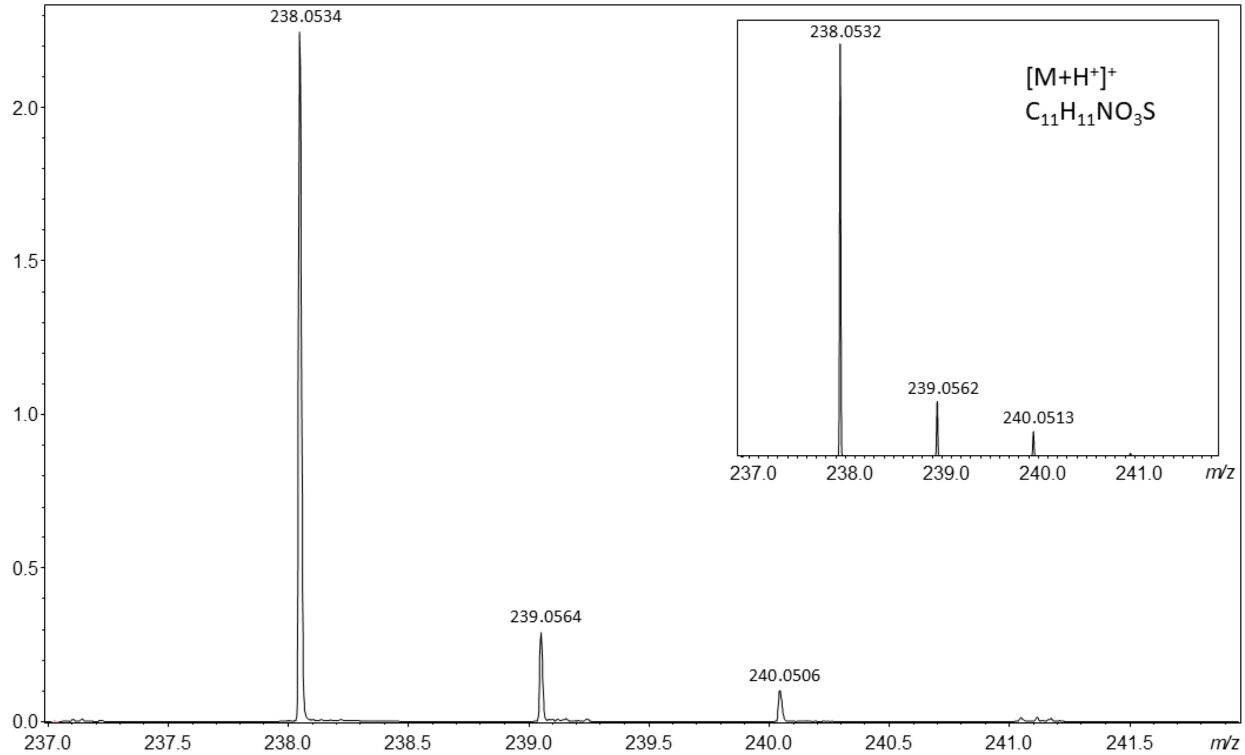

Supplementary figure 7. High-resolution mass spectrum of fraction P1 in Supplementary figure 5 showing experimental and simulated (inset) isotope distributions for 2-amino-3-(1-oxidobenzo[b]thiophen-3-yl)propanoic acid ($C_{11}H_{11}NO_3S$).

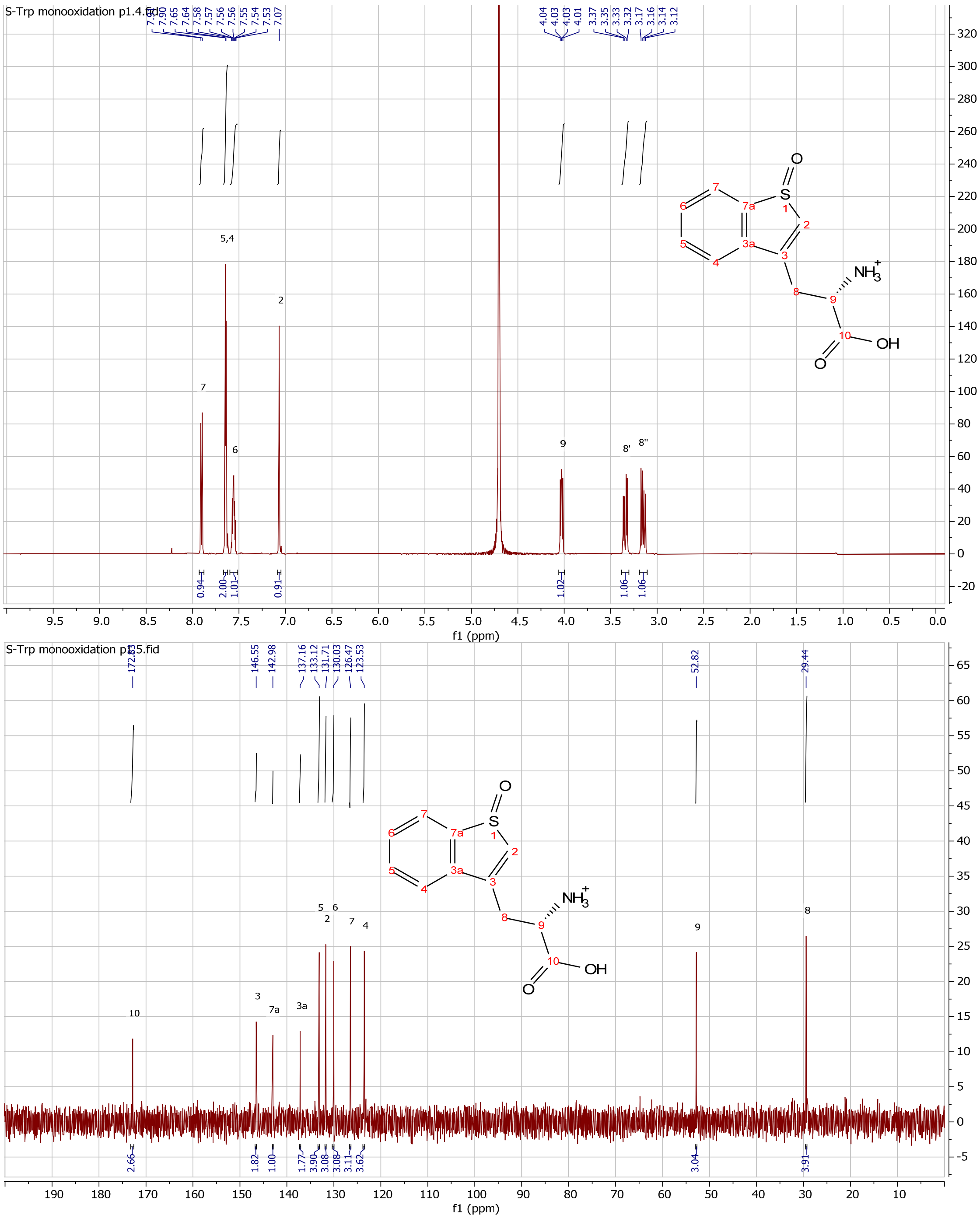

Supplementary figure 8. ^{1}H NMR (500 MHz, D_2O) and ^{13}C NMR (126 MHz, D_2O) spectra of fraction P1 in Supplementary figure 5.

^{1}H NMR (500 MHz, D_2O) δ 7.90 (d, J = 7.7 Hz, ^{1}H), 7.64 (d, J = 4.0 Hz, ^{2}H), 7.56 (dq, J = 8.3, 4.3 Hz, ^{1}H), 7.07 (s, ^{1}H), 4.03 (dd, J = 8.6, 5.4 Hz, ^{1}H), 3.34 (dd, J = 15.6, 5.4 Hz, ^{1}H), 3.15 (dd, J = 15.6, 8.6 Hz, ^{1}H). ^{13}C NMR (126 MHz, D_2O) δ 172.83, 146.55, 142.98, 137.16, 133.12, 131.71, 130.03, 126.47, 123.53, 52.82, 29.44.

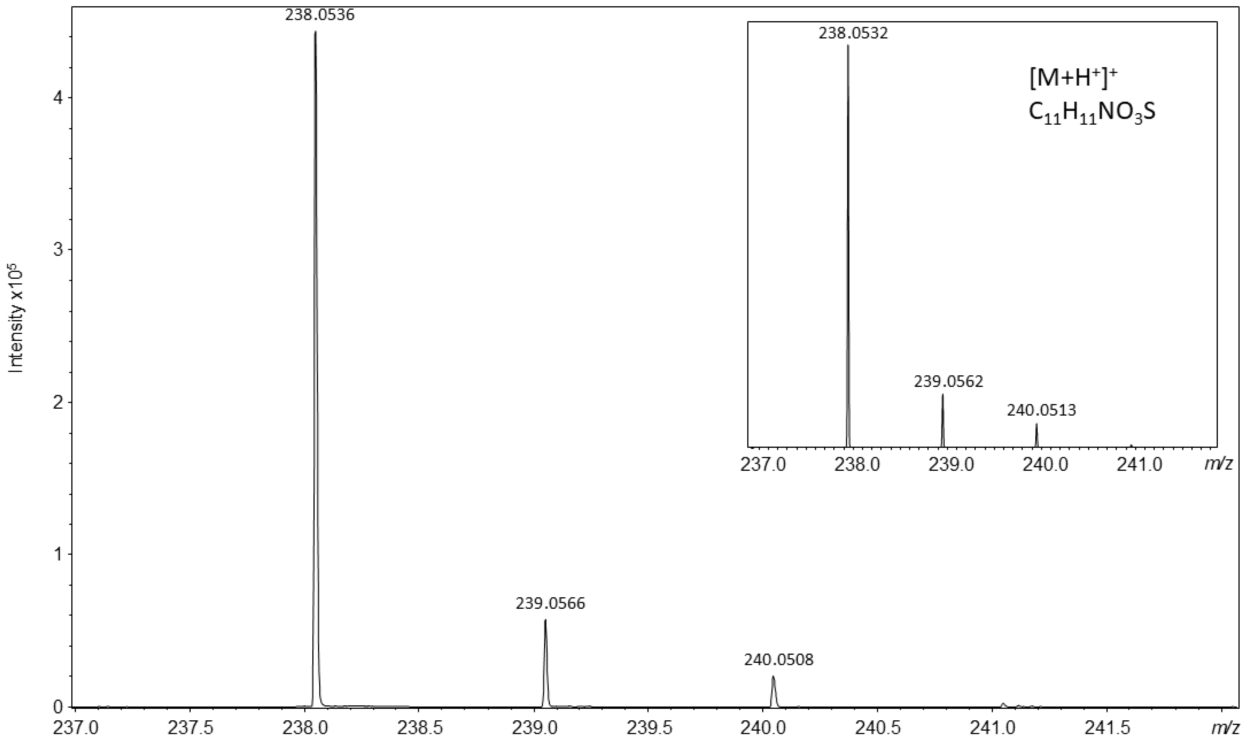

Supplementary figure 9. High-resolution mass spectrum of fraction P2 in Supplementary figure 5 showing experimental and simulated (inset) isotope distributions for 2-amino-3-(1-oxidobenzo[b]thiophen-3-yl)propanoic acid ($C_{11}H_{11}NO_3S$).

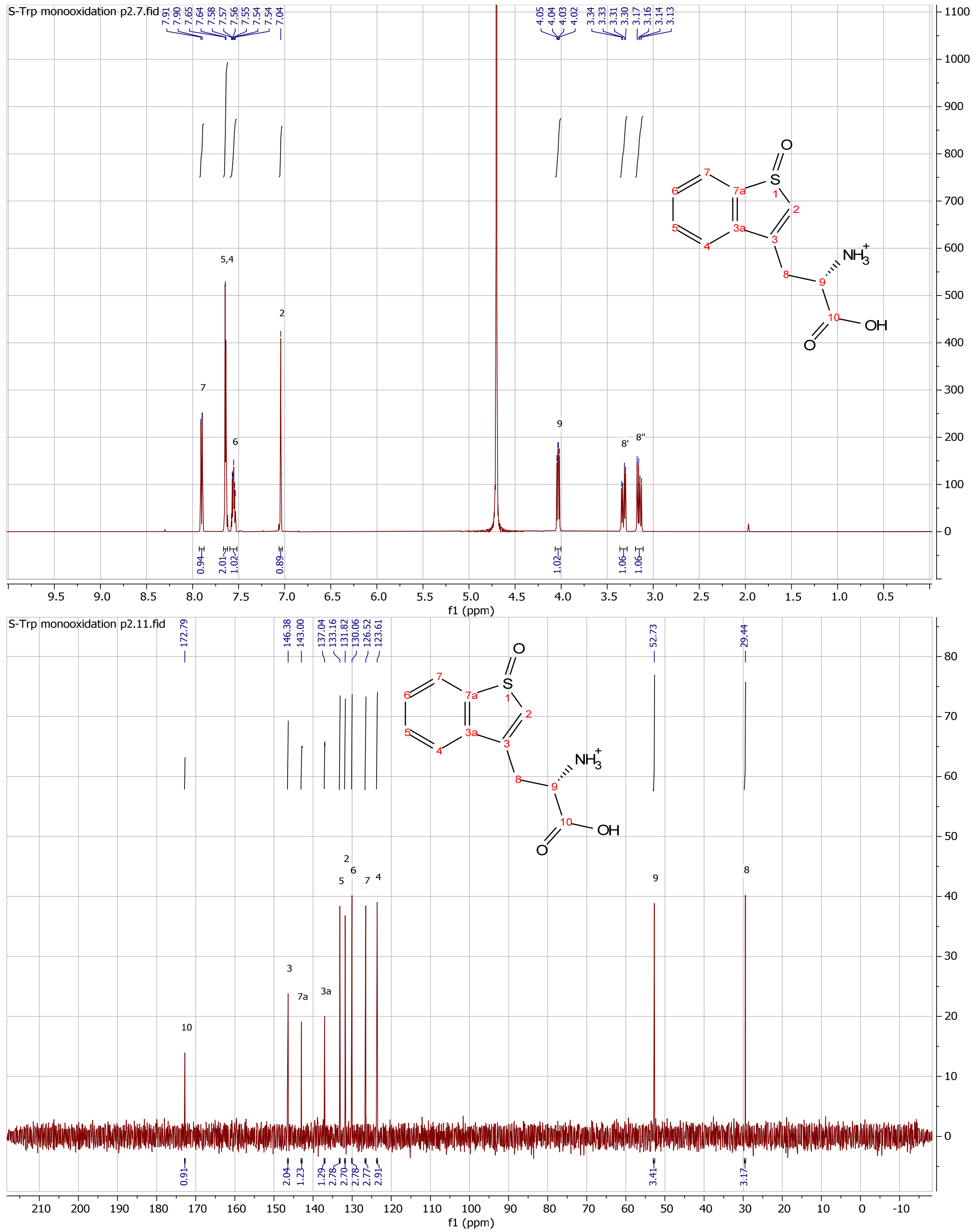

Supplementary figure 10. ^{1}H NMR (500 MHz, D_2O) and ^{13}C NMR (126 MHz, D_2O) spectra of fraction P2 in Supplementary figure 5.

^{1}H NMR (500 MHz, D_2O) δ 7.90 (dd, J = 7.5, 0.9 Hz, ^{1}H), 7.64 (d, J = 4.2 Hz, ^{2}H), 7.55 (dq, J = 8.3, 3.5 Hz, ^{1}H), 7.04 (s, ^{1}H), 4.03 (dd, J = 8.2, 5.6 Hz, ^{1}H), 3.32 (dd, J = 15.4, 5.6 Hz, ^{1}H), 3.15 (dd, J = 15.4, 8.2 Hz, ^{1}H).
^{13}C NMR (126 MHz, D_2O) δ 172.79, 146.38, 143.00, 137.04, 133.16, 131.82, 130.06, 126.52, 123.61, 52.73, 29.44.

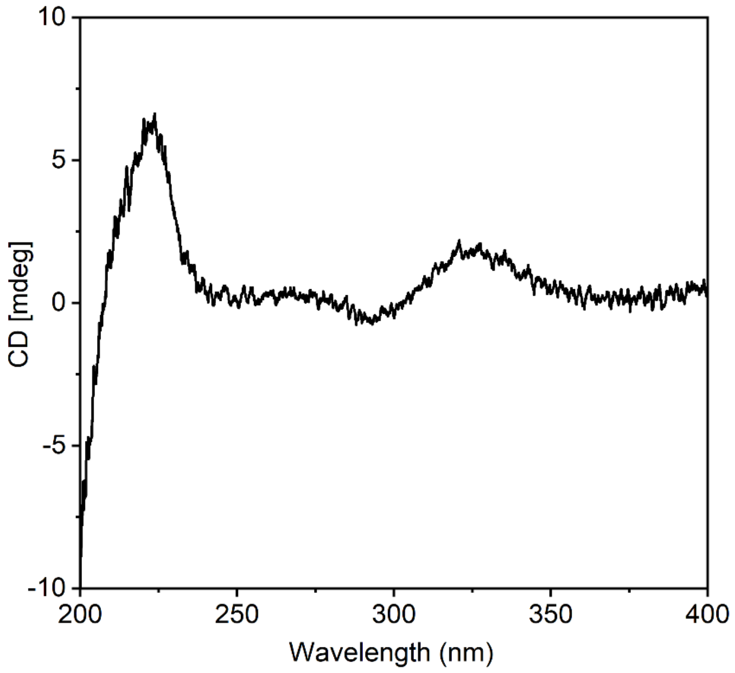

Supplementary figure 11. Original circular dichroism (CD) spectrum of the S-Trp105-bearing peptide isolated from digested KatG S-Trp105 via HPLC.

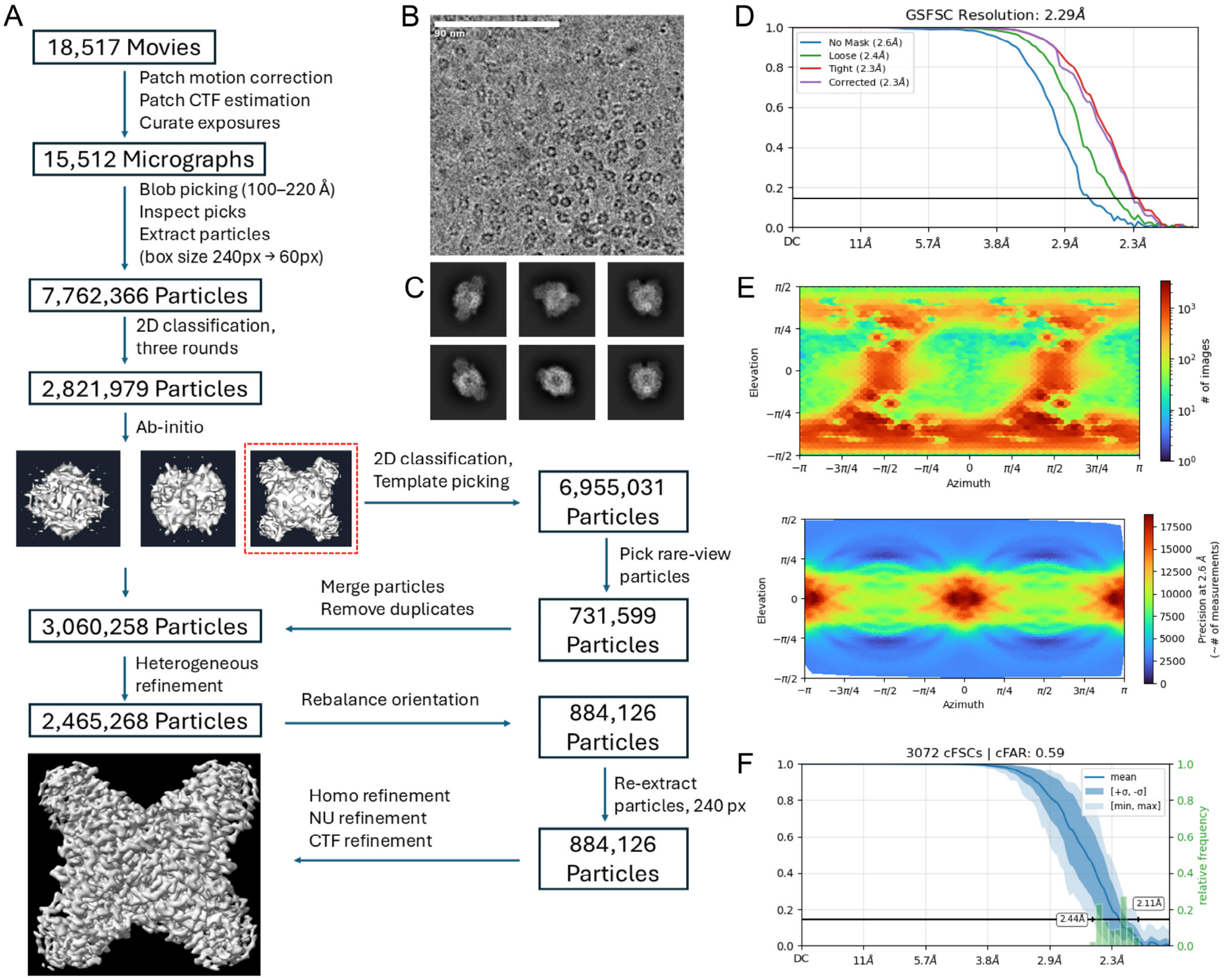

Supplementary figure 12. Cryo-EM data processing workflow. (A) A flow chart illustrates the cryo-EM data processing pipeline, (B) Representative cryo-EM micrograph, (C) Representative 2D class averages, (D) Gold-standard Fourier shell correlation (GSFSC) curves of the final reconstruction, (E) Viewing direction distribution and posterior precision directional distribution of particles used in the final reconstruction, and (F) Directional FSC plot of the final reconstruction.

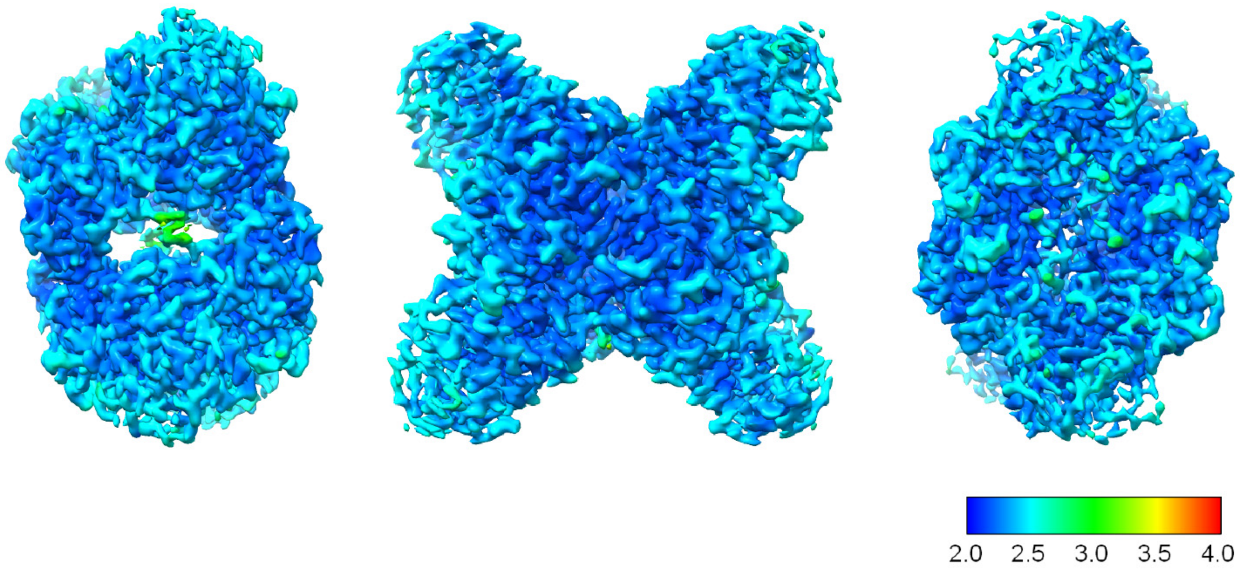

Supplementary figure 13. Local resolution of the cryo-EM density map. Local resolution map of the raw cryo-EM density map calculated using CryoSPARC and visualized with ChimeraX. Side, front, and top views are shown, with the resolution scale (in Å) at the bottom right.

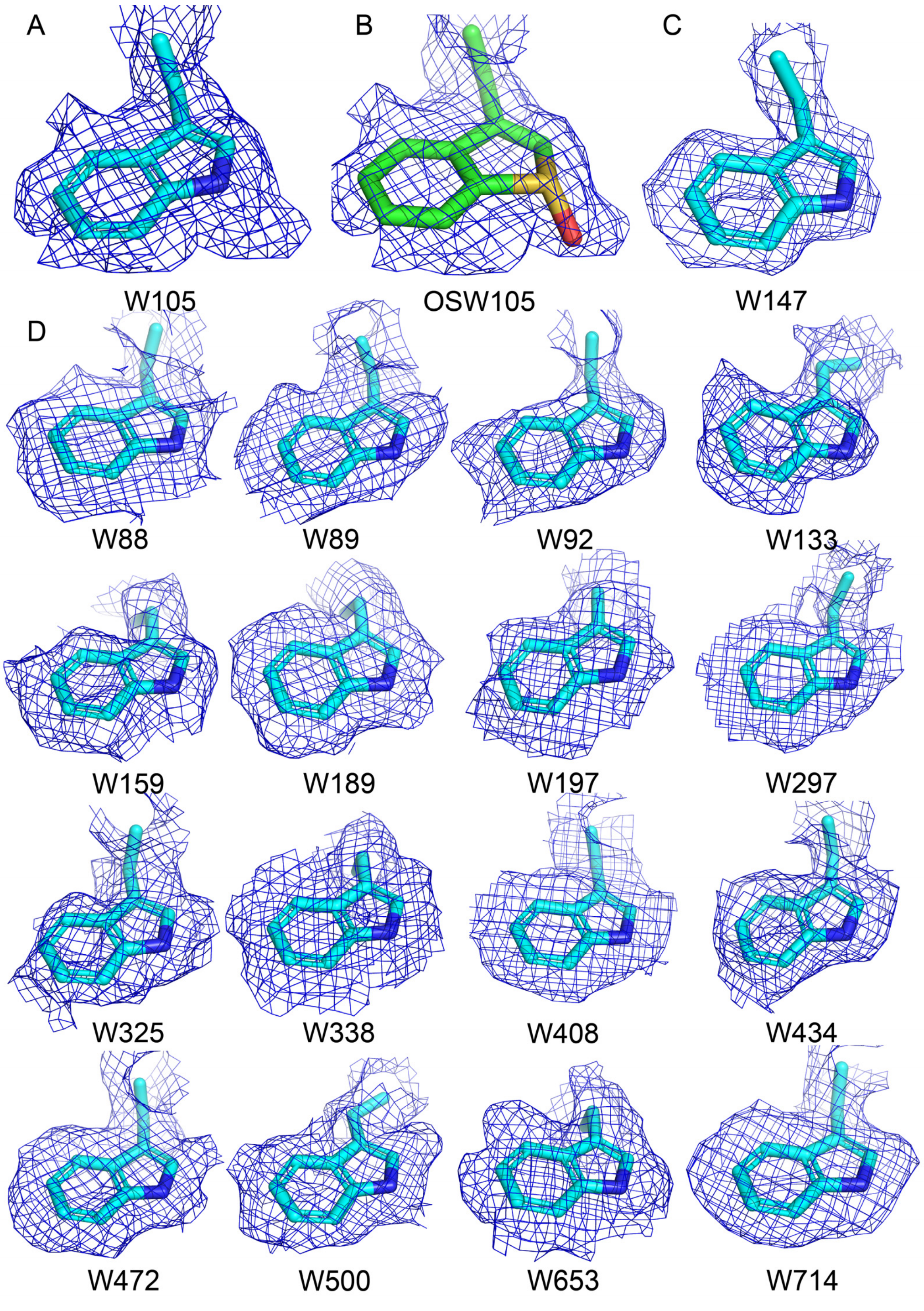

Supplementary figure 14. Comparison of density map and model fitting on monooxygenated S-Trp (molecular code in the structure: OSW) vs. unmodified Trp residues: (A) OSW105 density map fit with L-Trp; (B) OSW (W*) density map fit with OSW; (C) Density map and model for W147; (D) Density map and model for all other Trp residues in the chain A of KatG S-Trp105 protein.

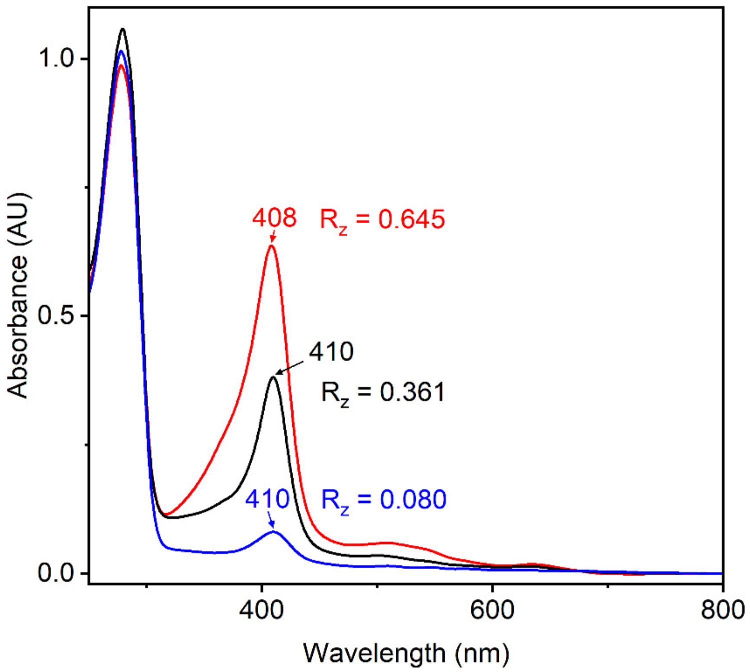

Supplementary figure 15. UV-vis and R_z value comparison of KatG S-Trp105, apo-KatG S-Trp105, and heme-reconstituted KatG S-Trp105. UV-vis spectra and corresponding R_z values are shown for as-isolated KatG S-Trp105 (black), apo-KatG S-Trp105 (blue), and heme-reconstituted KatG S-Trp105 (red).

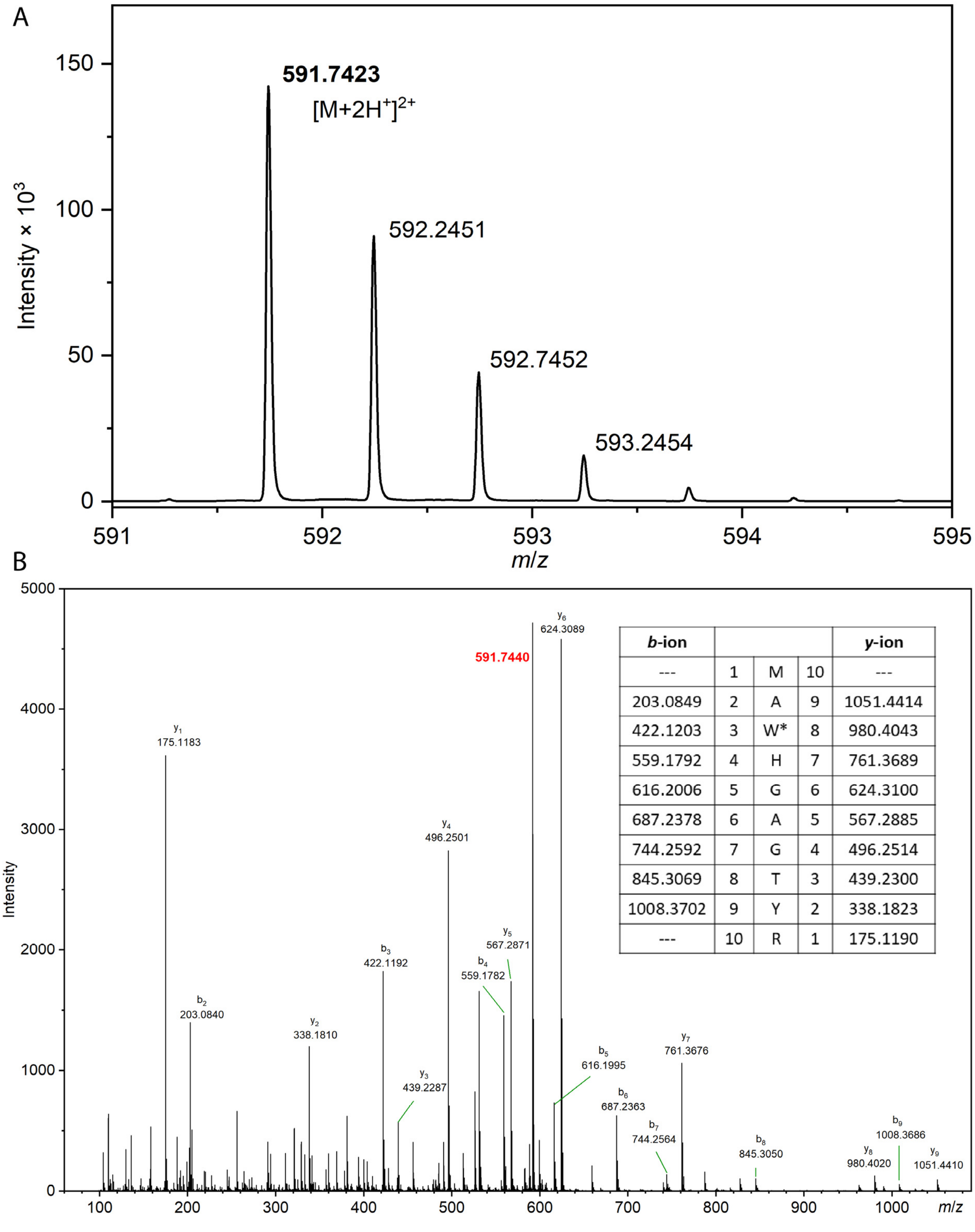

***b*-ion**				***y*-ion**
---	1	M	10	---
203.0849	2	A	9	1051.4414
422.1203	3	W*	8	980.4043
559.1792	4	H	7	761.3689
616.2006	5	G	6	624.3100
687.2378	6	A	5	567.2885
744.2592	7	G	4	496.2514
845.3069	8	T	3	439.2300
1008.3702	9	Y	2	338.1823
---	10	R	1	175.1190

Supplementary figure 16. HRMS spectrum (A) and CID spectrum (B) for regenerated O=S-Trp bearing peptide. The inset shows the fragment ion assignments.

Supplementary table 1. Fragment assignment for the CID spectrum in Fig. 3C

Residue formula for W*	$C_{11}H_9N_1O_2S_1$					
Predicted b-ions	Detected b-ions	b-ion	Residues	y-ion	Detected y-ions	Predicted y-ions
---		1	M	10		---
203.0849	203.0851	2	A	9	1051.4436	1051.4414
422.1203	422.1208	3	W*	8	980.4028	980.4043
559.1792	559.1800	4	H	7	761.3696	761.3689
616.2006	616.2013	5	G	6	624.3105	624.3100
687.2378	687.2385	6	A	5	567.2893	567.2885
744.2592	744.2599	7	G	4	496.2521	496.2514
845.3069	845.3064	8	T	3	439.2300	439.2300
1008.3702	1008.3702	9	Y	2	338.1826	338.1823
---		10	R	1	175.1190	175.119

The monooxygenated S-Trp residue was assigned as W*.
Theoretical masses for the b- and y-type fragment ions were calculated using the MS-Product utility of ProteinProspector (https://prospector.ucsf.edu/prospector/mshome.htm) using [$C_{11}H_9NO_2S$] for the monooxygenated S-Trp residue (W*).

Supplementary table 2. Cryo-EM data collection, processing, and refinement statistics (PDB DOI: https://doi.org/10.2210/pdb9O6A/pdb; EMDB: https://www.ebi.ac.uk/emdb/EMD-70168)

	S-Trp105 EcKatG
Deposited Files	EMDB-70168; PDB-9O6A
Data Collection and Processing	SSRL
Electron Microscope	Titan Krios G3i
Voltage (kV)	300 keV
Camera	Falcon 4i
Defocus range (μm)	-1.0 ~ -2.0
Total exposure time (s)	6.58
Energy filter width (eV)	10
Pixel size (Å) (calibrated)	0.954
Total dose (e-/Å2)	50
Number of frames	38
Does per frame (e^-/Å^2)	1.1357
Magnification (nominal)	130,000×
No. of initial micrographs	15512
No. of initial particles	7,762,366
No. of final particles	884,126
Symmetry	D2
Map Resolution (Å)	2.29
FSC threshold	0.143
Density modification Resolution (Å)	2.22
Refinement and Validation	
Map-sharping B-factors (Å2)	-7.92
Model composition	
Chain	4
Protein residues	2576
Ligands (heme)	4
r.m.s. deviationsa	
Bond lengthsd (Å)	0.003
Bond angles (°)	0.601592
MolProbity score	1.0819
Ramachandrane	
Favored (%)	98.4778
Allowed (%)	1.4918
Outlier (%)	0.04
ADP (B-factors) (min/max/mean) (Å^2)	
Protein	0.01/77.15/20.17
Ligand	8.43/29.45/16.19
CCb Mask	0.79

[a]root means square deviation
[b]correlation coefficient

Supplementary table 3. The stereochemical restraints of O=S-Trp during cryo-EM data processing. The restraints were established based on reported small-molecule structures of benzothiophene S-oxide analogs listed in the table.

3D structure	Bond length (Å)	Bond angle (°)	CCDC number	Reference
	S-C1	C1-S-C2		
	S-C2	C1-S-O		
	S-O	C2-S-O		
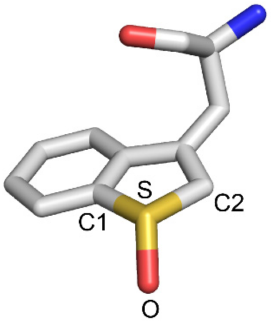	1.77<br>1.78<br>1.49	112.4<br>113.7<br>89.9	N/A	This work
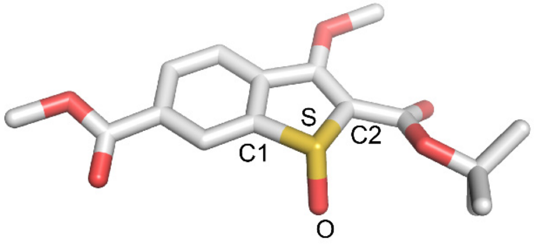	1.79<br>1.80<br>1.49	109.2<br>112.9<br>89.4	1842115	Ref. 1
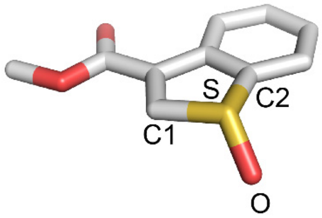	1.78<br>1.77<br>1.49	113.4<br>111.5<br>90.3	2236439	Ref. 2

The stereochemical restraints for S-monooxygenated S-Trp (OSW) are shown in a CIF file shown below.

The corresponding CIF File associated with Supplementary table 3:

```
data_comp_list
loop_
_chem_comp.id
_chem_comp.three_letter_code
_chem_comp.name
_chem_comp.group
_chem_comp.number_atoms_all
_chem_comp.number_atoms_nh
_chem_comp.desc_level
OSW       OSW 'Unknown                  ' ligand 15 15 .
#
data_comp_OSW
#
loop_
_chem_comp_atom.comp_id
_chem_comp_atom.atom_id
_chem_comp_atom.type_symbol
_chem_comp_atom.type_energy
_chem_comp_atom.charge
_chem_comp_atom.partial_charge
_chem_comp_atom.x
_chem_comp_atom.y
_chem_comp_atom.z
OSW       N     N   NH2    0    .     119.5940   84.6760  146.4100
OSW       CA    C   CH1    0    .     118.4470   84.4860  147.2410
OSW       C     C   C1     0    .     118.0770   85.7980  147.9700
OSW       O     O   O      0    .     116.9390   86.1590  148.0120
OSW       CB    C   CH2    0    .     118.6830   83.3670  148.2660
OSW       CG    C   CH1    0    .     118.0430   83.6800  149.6580
OSW       CD1   C   CH2    0    .     118.7890   83.9230  150.7400
OSW       CD2   C   CR6    0    .     116.5960   83.6100  149.9160
OSW       CE2   C   CR6    0    .     116.3310   83.8440  151.2730
OSW       CE3   C   CR16   0    .     115.5390   83.3480  149.0540
OSW       CZ2   C   CR16   0    .     115.0460   83.8240  151.7930
OSW       CZ3   C   CR16   0    .     114.2450   83.2850  149.5800
OSW       CH2   C   CR16   0    .     113.9990   83.5040  150.9370
OSW       O1    O   OH1    0    .     118.0340   83.4370  153.3300
OSW       S     S   SH1    0    .     117.7860   84.3250  152.1560
#
loop_
_chem_comp_bond.comp_id
_chem_comp_bond.atom_id_1
_chem_comp_bond.atom_id_2
_chem_comp_bond.type
```

```
_chem_comp_bond.value_dist
_chem_comp_bond.value_dist_esd
_chem_comp_bond.value_dist_neutron
OSW   N       CA    single       1.429 0.020     1.429
OSW   CA      C     single       1.546 0.020     1.546
OSW   CA      CB    single       1.536 0.020     1.536
OSW   C       O     double       1.195 0.020     1.195
OSW   CB      CG    single       1.564 0.020     1.564
OSW   CG      CD1   single       1.337 0.020     1.337
OSW   CG      CD2   single       1.471 0.020     1.471
OSW   CD1     S     single       1.781 0.020     1.781
OSW   CD2     CE2   aromatic     1.402 0.020     1.402
OSW   CD2     CE3   aromatic     1.389 0.020     1.389
OSW   CE2     CZ2   aromatic     1.386 0.020     1.386
OSW   CE2     S     single       1.769 0.020     1.769
OSW   CE3     CZ3   aromatic     1.398 0.020     1.398
OSW   CZ2     CH2   aromatic     1.390 0.020     1.390
OSW   CZ3     CH2   aromatic     1.396 0.020     1.396
OSW   O1      S     single       1.493 0.020     1.493
#
loop_
_chem_comp_angle.comp_id
_chem_comp_angle.atom_id_1
_chem_comp_angle.atom_id_2
_chem_comp_angle.atom_id_3
_chem_comp_angle.value_angle
_chem_comp_angle.value_angle_esd
OSW   CB      CA      C        109.90 3.000
OSW   CB      CA      N        111.20 3.000
OSW   C       CA      N        110.70 3.000
OSW   O       C       CA       120.07 3.000
OSW   CG      CB      CA       112.67 3.000
OSW   CD2     CG      CD1      114.56 3.000
OSW   CD2     CG      CB       123.30 3.000
OSW   CD1     CG      CB       121.91 3.000
OSW   S       CD1     CG       111.73 3.000
OSW   CE3     CD2     CE2      119.23 3.000
OSW   CE3     CD2     CG       130.43 3.000
OSW   CE2     CD2     CG       110.34 3.000
OSW   S       CE2     CZ2      125.39 3.000
OSW   S       CE2     CD2      111.90 3.000
OSW   CZ2     CE2     CD2      122.39 3.000
OSW   CZ3     CE3     CD2      118.64 3.000
OSW   CH2     CZ2     CE2      118.07 3.000
OSW   CH2     CZ3     CE3      121.44 3.000
OSW   CZ3     CH2     CZ2      120.13 3.000
```

```
OSW   O1      S       CE2         111.56 3.000
OSW   O1      S       CD1         113.42 3.000
OSW   CE2     S       CD1          90.29 3.000
#
loop_
_chem_comp_tor.comp_id
_chem_comp_tor.id
_chem_comp_tor.atom_id_1
_chem_comp_tor.atom_id_2
_chem_comp_tor.atom_id_3
_chem_comp_tor.atom_id_4
_chem_comp_tor.value_angle
_chem_comp_tor.value_angle_esd
_chem_comp_tor.period
OSW CONST_01      CH2     CZ2     CE2     CD2           2.74   0.0 0
OSW CONST_02      CH2     CZ3     CE3     CD2           1.23   0.0 0
OSW CONST_03      CZ3     CE3     CD2     CE2          -1.99   0.0 0
OSW CONST_04      CZ3     CH2     CZ2     CE2          -3.47   0.0 0
OSW CONST_05      CZ2     CE2     CD2     CE3           0.01   0.0 0
OSW CONST_06      CZ2     CH2     CZ3     CE3           1.56   0.0 0
OSW Var_01        CE2     S       CD1     CG           10.05  30.0 1
OSW Var_02        CE2     CD2     CG      CD1           1.02  30.0 1
OSW Var_03        CD2     CE2     S       CD1          -9.45  30.0 1
OSW Var_04        S       CD1     CG      CD2          -8.19  30.0 1
OSW Var_05        CD1     CG      CB      CA         -111.72  30.0 3
OSW Var_06        CG      CB      CA      N           142.24  30.0 3
OSW Var_07        O       C       CA      N           134.53  30.0 3
#
loop_
_chem_comp_chir.comp_id
_chem_comp_chir.id
_chem_comp_chir.atom_id_centre
_chem_comp_chir.atom_id_1
_chem_comp_chir.atom_id_2
_chem_comp_chir.atom_id_3
_chem_comp_chir.volume_sign
OSW chir_01   CA      N       C       CB    both
OSW chir_02   CG      CD2     CD1     CB    both
OSW chir_03   S       O1      CE2     CD1   both
#
loop_
_chem_comp_plane_atom.comp_id
_chem_comp_plane_atom.plane_id
_chem_comp_plane_atom.atom_id
_chem_comp_plane_atom.dist_esd
OSW plan-1     CA 0.020
```

OSW plan-1	C	0.020
OSW plan-1	O	0.020
OSW plan-2	CG	0.020
OSW plan-2	CD2	0.020
OSW plan-2	CE2	0.020
OSW plan-2	CE3	0.020
OSW plan-2	CZ2	0.020
OSW plan-2	CZ3	0.020
OSW plan-2	CH2	0.020
OSW plan-2	S	0.020

Supplementary table 4. Investigated potential heme-mediated autooxidation products in KatG S-Trp105

Met Tyr S-Trp	no crosslink
Met Tyr (O-Trp-S)	no crosslink, C-monooxygenation
Met Tyr O=S-Trp	no crosslink, S-monooxygenation
Met Tyr (Trp-S)O_2	S-dioxygenation
Met Tyr-(Trp-S)	partial crosslink
Met Tyr-(Trp-S=O)	partial crosslink
Met Tyr-(Trp-SO_2)	partial + dioxygenation
Met +-Tyr-(Trp-S)	full crosslink, no oxygenation
Met+-Tyr-(Trp-S)O	crosslink + S-oxygenation
Met+-Tyr-(Trp-S)O_2	crosslink + S-dioxygenation

References Cited:

1. Ndzeidze GN, Li L, Steinmetz MG. Reversible triplet excitation transfer in a trimethylene-linked thioxanthone and benzothiophene-2-carboxanilide that photochemically expels leaving group anions. *J Org Chem* **83**, 8995-9007 (2018).

2. Bisht R, Popescu MV, He Z, Ibrahim AM, Crisenza GEM, Paton RS, Procter DJ. Metal-free arylation of benzothiophenes at C4 by activation as their benzothiophene S-oxides. *Angew Chem Int Ed* **62**, e202302418 (2023).
