# Supplementary material for: Single-atom substitution redirects KatG reactivity from cofactor biogenesis to stereoselective sulfoxidation
Source: Nat Commun. 2026 Jun 13;17:5309. doi: 10.1038/s41467-026-73579-y (PMC13272816; doi:10.1038/s41467-026-73579-y)
Supplement: Supplementary file 3 — Transparent Peer Review file [file 41467_2026_73579_MOESM3_ESM.pdf]

nature portfolio

Peer Review File

Single-atom substitution redirects KatG reactivity from cofactor biogenesis to stereoselective sulfoxidation

Corresponding Author: Professor Aimin Liu

This file contains all reviewer reports in order by version, followed by all author rebuttals in order by version.

Version 0:

Reviewer comments:

Reviewer #1

(Remarks to the Author)
This paper looks at the unusual Met-Tyr-Trp cross-linked cofactor in a a catalase-peroxidase (KatG) enzyme. The paper reports the replacement of Trp105 of the M-Y-W triad with a S-Trp non-canonical amino acid. The authors demonstrate that the function of the enzyme switches from cofactor-forming to sulfoxide-forming reactivity when Trp is swapped with S-Trp. They have therefore discovered a pseudo-monooxygenase activity that is switched on in the enzyme merely by replacement of the two atoms of NH with one atom of S. The conclusions that are drawn – which are supported by mass spectrometry, cryo-EM and other spectroscopic data of very convincing quality – have profound relevance for understanding of heme protein reactivity. I am therefore very supportive of the paper, and have fairly minor comments.

- The most substantive point is the switch of reactivity that is facilitated by the Trp/S-Trp swap. There is a complete (?) switch off of the cofactor formation, and a complete (?) switch on of the monooxygenase activity. And the authors have thus confirmed that the NH group of Trp is essential for cofactor formation. And yet the title of the paper doesn't quite convey these concepts. It is not, to my reading, "catalytic switching through stereoselective sulfoxidation". It is catalytic switching TO stereoselective sulfoxidation FROM cofactor formation BY Trp swaps. I ask the authors to look at the title, and make sure that the main message isn't lost. Perhaps their phase at the end of the results "single atom substitution (at Trp105?) redirects KatG reactivity ….. from X to Y" better sums it up. But it is up to the authors to decide.

- At the end of the discussion, a mechanism is presented. All very sensible, but I did feel the paper needed a figure here so that the reader can easily compare the two reactivities that are being switched on/off. I wasn't sure that step 2 of Scheme 2 was enough as it doesn't show the proposed Compound I formation. Is it possible to draw out a simple scheme where the two reactivities are clearly shown, with the common steps (Compound I formation) shown? At the very least, formation of the sulphoxide needs to be shown in full (with the heme intermediates and some arrow pushing as well??).

- I also felt that the discussion of the Fe-O-S transition state needed a picture, perhaps as part of the figure above. With this and the other pictures above, the reader would easily be able to capture the relative reactivity of the Trp residues in the similar peroxidase and KatG enzymes.

- The authors draw out comparisons with the peroxidases in the Discussion. I agree with the authors (half way down p11) that CcP and APX do typically remain unmodified under normal turnover conditions. There is, however, evidence from a while back for radical formation on Trp 51 of CcP/mutants (JMB (2003) 328, 157; Biochemistry (2009) 48, 3593) and APX (Biochemistry (2007) 46, 2174) and covalent links, even to the heme, can be formed in some cases (e.g. Biochemistry (2007) 46, 13269). At the time this work was published, much less was known on KatG and the mass spec methods have moved on – so if the authors think this is still relevant, it would be good to know what their view is on the comparisons between KatG and these reported peroxidase reactivities.

- Formation of sulphoxides, during peroxidase-catalysed of (exogenous) sulphides, has been known for a long time. Different mechanism of course, but it might just be worth flagging some of this early work (Dunford or Paul Ortiz de Montellano may have reviewed it at some point), to make the direct comparison with substrate sulphoxidation.

- Minor issue that the last 4 lines of p3 seems to repeat themselves.

- Ditto the last 4 lines of p10 (“in the absence…..”).

- A very nice paper overall.

Reviewer #2

(Remarks to the Author)
This work shows that replacing tryptophan 105 in the enzyme KatG from E.coli by the unnatural amino acid thiotryptophan predictably leads to the loss of original catalytic function, but is surprisingly accompanied by an irreversible stereoselective autooxidation of thiotryptophan. Although this a cul-de-sac for the enzyme, the observation and characterization of the thiotryptophan sulfoxide is novel.

The multi-method experimental approach is comprehensive, the experiments are meticulously executed, and the penmanship is excellent.

Minor comments are listed below.

Page 5 line 13: "but functionally significant" - The slight change in the UV/vis spectrum does not support this conclusion about functional significance (although later data does). Rephrase.

Page 4 lines 23-26: "The magnitude and pattern..." - Again, the conclusion that reactivity is redirected is not directly supported by the activity assays - they just show reduced activity, not pointing to an "alternative reaction pathway". The next section does though. I suggest rephrasing.

Page 6 line 8: "alternative, site-specific oxidative modification" - from the HPLC and UV/vis data, it is not obvious why this would indicate an oxidative modification, rather than just the aromatic substitution. Clarify. (The following sentence is clearer in this regard).

Page 7 line 26: Unclear what "Those" refers to.

Page 7 line 29: Mtb undefined.

Page 9 line 11: Define what Rz is; this might be unfamiliar to many readers

Page 10 line 22: It is a bit of a stretch to call this autooxidation of a non-functional mutant "latent catalytic plasticity".

Page 11 line 13: 43 superscript

Reviewer #3

(Remarks to the Author)
The Authors present a study where genetic code expansion has been used to replace residue Trp105 of catalase/peroxidase KatG. This residue has previously been implicated in the formation of cross-links between methionine, tyrosine and tryptophan to form the MYW peptide that is essential for the catalase activity of KatG. The formation of cross-links is proposed to occur via a radical atom that relies on hydrogen atom transfer via the indole nitrogen of trp105. Replacing this atom with S in thiotryptophan removes the hydrogen atom that is believed to be transferred in an attempt to probe the early stages of cross-link formation. However, unexpected oxygenation of the sulfur atom occurred in the variant enzyme and the authors present structural and spectroscopic data to confirm formation of a sulfoxide on thiotrp 105 and also investigate the stereochemistry of the product and show it's formation is dependent on the presenece of haem and peroxide.

I found the paper to be well written and the evidence presented to be largely compelling and fully supportive of the conclusions drawn. The cryoEM structure clearly showed the modification of Trp105 and the spectroscopy largely supported this (see comment below).

I have a few minor comments, which are more points of curiosity rather than criticisms of the work presented.

I found the substitution of trptophan with thiotryptophan an interesting choice, clearly there is a precedent for this in the literature but I wondered if there was any specific reason why the indole ring was not replaced with a benzofuran where the N is replaced by O? This should retain the chemical characteristics of thiotryptptphan (aromaticity etc) but adds a heterotaom that only differs by one proton/electron. Given the general readership of nature comms I think a comment in the introduction would be appropriate.

Also are there plans to attempt the same experiment but replacing the N with P (phosphoindole) which would replace N with

an atom of the same group but with different protonation/deprotonation behaviour?

Addition of a reaction mechanism for the proposed 2-electron oxygen atom transfer similar to the scheme S1 would be a nice addition.

In figure 3 there is no indication of the peak heights in the FTIR, could this be added? Also the blue trace (free O=S-trp) is very noisy and not very convincing. The diagnostic peak is similar size to what are presumably noise peaks.

In figure 4 the legend should state the 2.22 A resolution is density modified and the resolution of the raw amps is 2.29 A. Showing density for an unmodified trp would also be a useful for comparison to that of O=S-trp.

If these comments can be addressed I have no hesitation in recommending this manuscript for submission.

Open Access This Peer Review File is licensed under a Creative Commons Attribution 4.0 International License, which permits use, sharing, adaptation, distribution and reproduction in any medium or format, as long as you give appropriate credit to the original author(s) and the source, provide a link to the Creative Commons license, and indicate if changes were made.
In cases where reviewers are anonymous, credit should be given to 'Anonymous Referee' and the source.
The images or other third party material in this Peer Review File are included in the article's Creative Commons license, unless indicated otherwise in a credit line to the material. If material is not included in the article's Creative Commons license and your intended use is not permitted by statutory regulation or exceeds the permitted use, you will need to obtain permission directly from the copyright holder.
To view a copy of this license, visit https://creativecommons.org/licenses/by/4.0/

NCOMMS-26-016386 - Point-by-Point Response to the Reviewers' Comments

Reviewer #1 (Remarks to the Author):

This paper looks at the unusual Met-Tyr-Trp cross-linked cofactor in a a catalase-peroxidase (KatG) enzyme. The paper reports the replacement of Trp105 of the M-Y-W triad with a S-Trp non-canonical amino acid. The authors demonstrate that the function of the enzyme switches from cofactor-forming to sulfoxide-forming reactivity when Trp is swapped with S-Trp. They have therefore discovered a pseudo-monooxygenase activity that is switched on in the enzyme merely by replacement of the two atoms of NH with one atom of S. The conclusions that are drawn – which are supported by mass spectrometry, cryo-EM and other spectroscopic data of very convincing quality – have profound relevance for understanding of heme protein reactivity. I am therefore very supportive of the paper, and have fairly minor comments.

We thank the reviewer for the very positive comments.

- The most substantive point is the switch of reactivity that is facilitated by the Trp/S-Trp swap. There is a complete (?) switch off of the cofactor formation, and a complete (?) switch on of the monooxygenase activity. And the authors have thus confirmed that the NH group of Trp is essential for cofactor formation. And yet the title of the paper doesn't quite convey these concepts. It is not, to my reading, "catalytic switching through stereoselective sulfoxidation". It is catalytic switching TO stereoselective sulfoxidation FROM cofactor formation BY Trp swaps. I ask the authors to look at the title, and make sure that the main message isn't lost. Perhaps their phase at the end of the results "single atom substitution (at Trp105?) redirects KatG reactivity ….. from X to Y" better sums it up. But it is up to the authors to decide.

We thank the reviewer for this insightful suggestion. We agree that "redirects" more accurately captures the fundamental shift in chemical outcome. We have revised the title to: **"Single-atom substitution redirects KatG reactivity from cofactor biogenesis to stereoselective sulfoxidation."** As the reviewer noted, the switch is indeed complete; we observe no MYW cofactor formation in the as-isolated S-Trp KatG, nor during in vitro reconstitution experiments.

- At the end of the discussion, a mechanism is presented. All very sensible, but I did feel the paper needed a figure here so that the reader can easily compare the two reactivities that are being switched on/off. I wasn't sure that step 2 of Scheme 2 was enough as it doesn't show the proposed Compound I formation. Is it possible to draw out a simple scheme where the two reactivities are clearly shown, with the common steps (Compound I formation) shown? At the very least, formation of the sulphoxide needs to be shown in full (with the heme intermediates and some arrow pushing as well??).

- I also felt that the discussion of the Fe-O-S transition state needed a picture, perhaps as part of the figure above. With this and the other pictures above, the reader would easily be able to capture the relative reactivity of the Trp residues in the similar peroxidase and KatG enzymes.

We have refined **Scheme 2** to include detailed arrow pushing that explicitly illustrates the two-electron nature of the sulfoxidation pathway. In Path B, we show the concerted but asynchronous transfer where one electron from the ferryl-oxo quenches the porphyrin pi-cation radical, while the sulfur lone pair initiates an attack on the iron-bound oxygen. This provides a clear electronic rationale for why the transition state is assigned as an Fe(IV) species with a single Fe-O bond at one side and a dashed bond between the S and O. This mechanistic detail

contrasts with the one-electron radical propagation seen in the native Path A. This distinguishes the two-electron OAT in the S-Trp variant from the one-electron HAT steps in the native enzyme.

- The authors draw out comparisons with the peroxidases in the Discussion. I agree with the authors (half way down p11) that CcP and APX do typically remain unmodified under normal turnover conditions. There is, however, evidence from a while back for radical formation on Trp 51 of CcP/mutants (JMB (2003) 328, 157; Biochemistry (2009) 48, 3593) and APX (Biochemistry (2007) 46, 2174) and covalent links, even to the heme, can be formed in some cases (e.g. Biochemistry (2007) 46, 13269). At the time this work was published, much less was known on KatG and the mass spec methods have moved on – so if the authors think this is still relevant, it would be good to know what their view is on the comparisons between KatG and these reported peroxidase reactivities.

We appreciate the reviewer pointing out these critical precedents. The radical formation on Trp51 in CcP and the covalent heme-links in APX mutants demonstrate the high reactivity of these distal tryptophans when the protein scaffold is perturbed. These cases underscore the unique "evolutionary choice" of KatG to use this reactivity for beneficial cofactor biogenesis rather than deleterious off-pathway modifications. We have added a discussion of these precedents to the manuscript, noting that while one-electron chemistry is seen in CcP/APX mutants, the redirection to a two-electron oxygenation in our KatG S-Trp variant represents a distinct catalytic "switch" revealed by single-atom precision. Additionally, we have compared a recent report of human indoleamine 2,3-dioxygenase (IDO), which can take free S-Trp as an alternate substrate and inserts one oxygen into its carbon but not sulfur. We added a discussion (the second last paragraph of the Discussion section) and cited these references on page 12.

- Formation of sulphoxides, during peroxidase-catalysed of (exogenous) sulphides, has been known for a long time. Different mechanism of course, but it might just be worth flagging some of this early work (Dunford or Paul Ortiz de Montellano may have reviewed it at some point), to make the direct comparison with substrate sulphoxidation.

We have included citations to the seminal work by Dunford and Ortiz de Montellano regarding peroxidase-catalyzed sulfoxidation of exogenous sulfides. We note that while substrate sulfoxidation is a known peroxidase capability, our work demonstrates the first instance of an endogenous residue being co-opted for this chemistry via genetic code expansion, transforming the enzyme's internal life cycle.

- Minor issue that the last 4 lines of p3 seems to repeat themselves.

- Ditto the last 4 lines of p10 ("in the absence…..").

- A very nice paper overall.

We have corrected the repetitive text on pages 3 and 10. We thank the reviewer for their careful proofreading.

Reviewer #2 (Remarks to the Author):

This work shows that replacing tryptophan 105 in the enzyme KatG from E.coli by the unnatural amino acid thiotryptophan predictably leads to the loss of original catalytic function, but is surprisingly accompanied by an irreversible stereoselective autooxidation of thiotryptophan.

Although this a cul-de-sac for the enzyme, the observation and characterization of the thiotryptophan sulfoxide is novel.

The multi-method experimental approach is comprehensive, the experiments are meticulously executed, and the penmanship is excellent.

We appreciate the above positive and encouraging summary.

Minor comments are listed below.

Page 5 line 13: "but functionally significant" - The slight change in the UV/vis spectrum does not support this conclusion about functional significance (although later data does). Rephrase.

We agree that UV-vis data alone do not establish functional significance. We have rephrased this to: "...induces subtle but detectable perturbations of the heme electronic environment (where the substituted word is underlined to replace "functionally significant" in the original text).

Page 4 lines 23-26: "The magnitude and pattern..." - Again, the conclusion that reactivity is redirected is not directly supported by the activity assays - they just show reduced activity, not pointing to an "alternative reaction pathway". The next section does though. I suggest rephrasing.

We have tempered this conclusion to better reflect the logical flow of the manuscript. The text now states: "The magnitude and pattern of these changes are consistent with the effective suppression of MYW-dependent catalysis, while the specific retention of residual peroxidase activity prompted our investigation into the alternative reaction pathway characterized in the following section.

Page 6 line 8: "alternative, site-specific oxidative modification" - from the HPLC and UV/vis data, it is not obvious why this would indicate an oxidative modification, rather than just the aromatic substitution. Clarify. (The following sentence is clearer in this regard).

We agree that the term "oxidative" jumps ahead of the evidence provided by mass spectrometry. We have revised this to: "...toward an alternative, site-specific chemical modification" (where "oxidative" has been replaced).

Page 7 line 26: Unclear what "Those" refers to.

We have clarified this sentence to specify that it refers to the broader set of variants explored in literature. It now reads: "Previously reported KatG variants lacking the MYW triad exhibit ..."

Page 7 line 29: Mtb undefined.

Mtb is now defined on page 3. We appreciate catching this oversight.

Page 9 line 11: Define what Rz is; this might be unfamiliar to many readers

R_z is now defined in the revised text.

Page 10 line 22: It is a bit of a stretch to call this autooxidation of a non-functional mutant "latent catalytic plasticity".

We understand the reviewer's point that this modification represents a loss of the enzyme's primary biological function. However, the high degree of enantioselectivity observed for the sulfoxidation—driven by the specific orientation of the residue relative to the heme—suggests a sophisticated, active site-mediated transformation rather than a stochastic autooxidation. To address the reviewer's concern, we have revised "latent catalytic plasticity" to "alternative chemical capability" to more accurately describe this reprogrammed reactivity.

Page 11 line 13: 43 superscript

Response: We have reformatted the reference citation to superscript.

Reviewer #3 (Remarks to the Author):

The Authors present a study where genetic code expansion has been used to replace residue Trp105 of catalase/peroxidase KatG. This residue has previously been implicated in the formation of cross-links between methionine, tyrosine and tryptophan to form the MYW peptide that is essential for the catalase activity of KatG. The formation of cross-links is proposed to occur via a radical atom that relies on hydrogen atom transfer via the indole nitrogen of trp105. Replacing this atom with S in thiotryptophan removes the hydrogen atom that is believed to be transferred in an attempt to probe the early stages of cross-link formation. However, unexpected oxygenation of the sulfur atom occurred in the variant enzyme and the authors present structural and spectroscopic data to confirm formation of a sulfoxide on thiotrp 105 and also investigate the stereochemistry of the product and show it's formation is dependent on the presenece of haem and peroxide.

I found the paper to be well written and the evidence presented to be largely compelling and fully supportive of the conclusions drawn. The cryoEM structure clearly showed the modification of Trp105 and the spectroscopy largely supported this (see comment below).

We thank the reviewer for their positive and supportive comments. We are pleased that the reviewer found our structural and spectroscopic evidence compelling and the paper well-written.

I have a few minor comments, which are more points of curiosity rather than criticisms of the work presented.

I found the substitution of trptophan with thiotryptophan an interesting choice, clearly there is a precedent for this in the literature but I wondered if there was any specific reason why the indole ring was not replaced with a benzofuran where the N is replaced by O? This should retain the chemical characteristics of thiotryptphan (aromaticity etc) but adds a heterotaom that only differs by one proton/electron. Given the general readership of nature comms I think a comment in the introduction would be appropriate.

Also are there plans to attempt the same experiment but replacing the N with P (phosphoindole) which would replace N with an atom of the same group but with different protonation/deprotonation behaviour?

We appreciate these forward-thinking suggestions regarding the scope of genetic code expansion. We chose thiotryptophan (S-Trp) specifically because sulfur's unique redox-sensitivity enables the two-electron oxygen-atom transfer (OAT) that is central to this study—a property that the oxygen analog (benzofuran) would likely lack, Also, L-3-(3-benzofuranyl)-alanine has limited availability.

Our engineered PylRS is specific that it excludes the native Trp, indicating that it has achieved a high level of stereoelectronic complementarity for the sulfur atom. However, developing a new high-fidelity pyrrolysyl-tRNA synthetase (PylRS) for another atomic analog is a non-trivial challenge. Our current evolved PylRS/$tRNA_{CUA}$ pair exhibits exceptional orthogonality, strictly discriminating against the native indole nitrogen to achieve high stereoelectronic complementarity for the sulfur atom. As added to the text: "The PylRS active site is precisely tuned to the unique bond lengths and polarizability of its cognate ncAA (S-Trp), highlighting the single-atom precision of this approach." The significantly smaller nitrogen (approx. 71 pm) or oxygen (approx. 66 pm) analogs than S-Trp (approx. 105 pm covalent radius) likely fail to establish the requisite steric contacts and induced-fit interactions necessary for efficient aminoacetylation. At this time, no PylRS is available for incorporating O-Trp or P-Trp.

Addition of a reaction mechanism for the proposed 2-electron oxygen atom transfer similar to the scheme S1 would be a nice addition.

This is also suggested by Reviewer 1. The revised Scheme 2 has addressed this need.

In figure 3 there is no indication of the peak heights in the FTIR, could this be added? Also the blue trace (free O=S-trp) is very noisy and not very convincing. The diagnostic peak is similar size to what are presumably noise peaks.

We have updated Figure 3 to explicitly indicate that the Y-axis represents normalized absorbance. Regarding the blue trace (free O=S-Trp), it is worth mentioning that this pure amino acid sample is in a solid crystalline state, and therefore its peak intensity cannot be directly compared with that of a liquid sample (DMSO) or a lyophilized protein. While it exhibits some background features due to the high concentration required for detection in the ATR-FTIR module, the diagnostic S=O stretching vibration at 1,041 cm^{-1} is prominent and reproducible. This feature is clearly mirrored by the 1,036 cm^{-1} peak in the KatG S-Trp105 variant (red trace) and is entirely absent in the WT enzyme (black trace), providing definitive spectroscopic evidence for sulfoxide formation.

In figure 4 the legend should state the 2.22 A resolution is density modified and the resolution of the raw amps is 2.29 A. Showing density for an unmodified trp would also be a useful for comparison to that of O=S-trp.

We have updated the legend for Figure 4 to clarify the resolution. It now reads: "(A) Cryo-EM map and atomic model of tetrameric KatG S-Trp105 at 2.22 Å resolution (density modified; raw map resolution 2.29 Å)".

Following the reviewer's suggestion, we have updated the original Fig. S13 that shows O=S-Trp in all four chains with a density map that attempting to fit by a Trp compared to O=S-Trp, and moved to the main text as Figure 5 for the sake of clarity (see on the next page). We replaced Fig. S13 with a side-by-side comparison of the density for the modified O=S-Trp105 against all native tryptophan residues elsewhere in the same KatG variant structure. This clearly illustrates

the additional, asymmetric density at position 105 that characterizes the sulfoxide modification. This is a brilliant move to add such a comparison. We appreciate reviewer's suggestions.

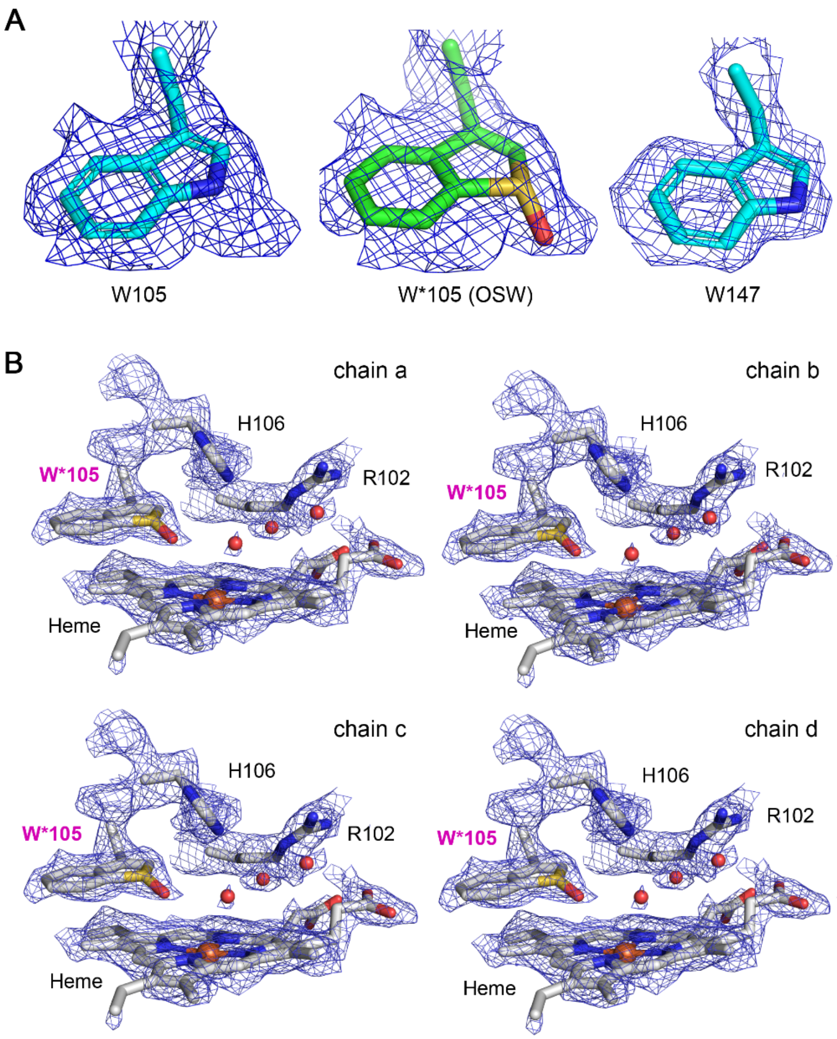

Figure 5. Stereogenic center of O=S-Trp105 in the cryo-EM structure of the engineered KatG variant. (A) Comparison of density map and model fitting on unmodified Trp, monooxygenated S-Trp (W*, molecular code in the 3D structure: OSW), and Trp147. Fig. S13 shows the comparison of O=S-Trp electron density with all other Trp residues in the same KatG structure. (B) The S-configured aromatic sulfur atom-centered configuration is uniformly observed in all four chains of the asymmetric unit. The cryo-EM density map contoured at 4.5 σ (ChimeraX).

If these comments can be addressed I have no hesitation in recommending this manuscript for submission.

Response: We appreciate your support.
